# Low-cost and clinically applicable copy number profiling using repeat DNA

**DOI:** 10.1186/s12864-022-08681-8

**Published:** 2022-08-17

**Authors:** Sam Abujudeh, Sebastian S. Zeki, Meta C.J. van Lanschot, Mark Pusung, Jamie M.J. Weaver, Xiaodun Li, Ayesha Noorani, Andrew J. Metz, Jan Bornschein, Lawrence Bower, Ahmad Miremadi, Rebecca C. Fitzgerald, Edward R. Morrissey, Andy G. Lynch

**Affiliations:** 1 Cancer Research UK Cambridge Institute, University of Cambridge, Li Ka Shing Centre, Robinson Way, Cambridge, CB2 0RE UK; 2 Medical Research Council (MRC) Cancer Unit, University of Cambridge, Cambridge, UK; 3 Department of Gastroenterology, Guy’s and St Thomas’ NHS Trust, London, SE1 7EH UK; 4 Department of Medical Oncology, The Christie NHS Foundation Trust, Manchester, M20 4TX UK; 5 Weatherall Institute of Molecular Medicine, University of Oxford, Oxford, UK; 6 School of Mathematics and Statistics/School of Medicine, University of St Andrews, St Andrews, UK

**Keywords:** Somatic copy number alterations, Copy number profiling, Cancer, Oesophageal adenocarcinoma, Barrett’s oesophagus, Tumour purity, FAST-SeqS, Bayesian nonparametrics, Probabilistic model, Sticky HDP-HMM, MCMC

## Abstract

**Background:**

Somatic copy number alterations (SCNAs) are an important class of genomic alteration in cancer. They are frequently observed in cancer samples, with studies showing that, on average, SCNAs affect 34% of a cancer cell’s genome. Furthermore, SCNAs have been shown to be major drivers of tumour development and have been associated with response to therapy and prognosis. Large-scale cancer genome studies suggest that tumours are driven by somatic copy number alterations (SCNAs) or single-nucleotide variants (SNVs). Despite the frequency of SCNAs and their clinical relevance, the use of genomics assays in the clinic is biased towards targeted gene panels, which identify SNVs but provide limited scope to detect SCNAs throughout the genome. There is a need for a comparably low-cost and simple method for high-resolution SCNA profiling.

**Results:**

We present conliga, a fully probabilistic method that infers SCNA profiles from a low-cost, simple, and clinically-relevant assay (FAST-SeqS). When applied to 11 high-purity oesophageal adenocarcinoma samples, we obtain good agreement (Spearman’s rank correlation coefficient, *r*_*s*_=0.94) between conliga’s inferred SCNA profiles using FAST-SeqS data (approximately £14 per sample) and those inferred by ASCAT using high-coverage WGS (gold-standard). We find that conliga outperforms CNVkit (*r*_*s*_=0.89), also applied to FAST-SeqS data, and is comparable to QDNAseq (*r*_*s*_=0.96) applied to low-coverage WGS, which is approximately four-fold more expensive, more laborious and less clinically-relevant. By performing an in silico dilution series experiment, we find that conliga is particularly suited to detecting SCNAs in low tumour purity samples. At two million reads per sample, conliga is able to detect SCNAs in all nine samples at 3% tumour purity and as low as 0.5% purity in one sample. Crucially, we show that conliga’s hidden state information can be used to decide when a sample is abnormal or normal, whereas CNVkit and QDNAseq cannot provide this critical information.

**Conclusions:**

We show that conliga provides high-resolution SCNA profiles using a convenient, low-cost assay. We believe conliga makes FAST-SeqS a more clinically valuable assay as well as a useful research tool, enabling inexpensive and fast copy number profiling of pre-malignant and cancer samples.

**Supplementary Information:**

The online version contains supplementary material available at (10.1186/s12864-022-08681-8).

## Background

Somatic copy number alterations (SCNAs) are common in cancer [1–5]. Certain SCNAs, particularly amplifications of oncogenes and deletions of tumour suppressor genes, have been found to be major drivers in tumour development, associated with prognosis and response to therapy [1, 6]. An early survey across multiple cancers saw SCNAs in 34% of the genome, with 17% of the genome amplified and 16% deleted [1], but SCNA burden varies considerably between cancer types [3, 5] (with, for example, thyroid cancer having low burden and ovarian cancer high burden) and so any average will depend on the mix of cancer samples considered.

Oesophageal adenocarcinoma (OAC) has relatively high levels of SCNAs [7–10], and generally develops from Barrett’s oesophagus (BO). Patients with OAC tend to be diagnosed at a late stage, when spread has occurred to lymph nodes and distant organs. This makes treatment more difficult and leads to poor prognosis [11]. Although most patients with BO do not progress, early-stage disease (high-grade dysplasia or intramucosal adenocarcinoma) can be successfully treated, usually obviating the need for surgery. There is a critical need to develop technologies that can detect early disease and distinguish between patients at low versus high risk for progression. Since most mutations in OAC driver genes are already present in pre-malignant disease [12], but an increased SCNA load can identify OAC [13–15], low-cost SCNA profiling would be a valuable research and clinical tool.

SCNAs have been identified using a number of methods, including comparative genomic hybridization (CGH) [16], array-based CGH [17], single-nucleotide polymorphism (SNP) arrays [18], targeted resequencing [19, 20] and whole-genome sequencing (WGS) [21]. Recently, low-coverage (LC) WGS has gained popularity due to its reduced cost and strong performance [22]. However, while LC WGS reduces the cost of sequencing, standard WGS library preparation is required with its associated fixed expense and time needed to produce each sample. A technically simple, fast, easily automated, high-resolution and inexpensive alternative method for SCNA detection, with clinical potential, would be extremely valuable.

Recent studies have shown the genome can be amplified at multiple (>10,000) genomic loci with the use of a single non-specific primer pair, using the FAST-SeqS method [23, 24]. With this approach, two polymerase chain reaction (PCR) rounds replace the complicated and expensive library preparation steps associated with WGS. The amplified regions are sufficiently short such that the assay can be performed on cell-free DNA as well as DNA extracted from tissue biopsies. The resulting amplicons can be sequenced, with samples multiplexed on the same sequencing lane. With this method, we maintain a similar sequencing depth to 30-50X high-coverage (HC) WGS while sequencing only specific loci. This is in contrast to LC WGS, which samples the whole genome but at reduced sequencing depth (Supplementary Fig. S1). We found the cost involved in sample preparation and sequencing combined to be approximately £14 per sample compared with approximately £52-72 for LC WGS, but this depends on the library preparation kit used (Supplementary Note 1). The sample preparation can be performed in less than an hour with minimal hands-on time, compared to approximately three hours or greater for LC WGS.

The use of FAST-SeqS data, until now, has largely been limited to the detection of whole chromosome gains [23] and entire chromosome arm gains and losses [24, 25]. This means that shorter chromosome segment (focal) alterations are not detected, or perhaps falsely considered as whole chromosome or chromosome arm alterations. Moreover, in these methods SCNAs are not quantified and regions are simply classified as amplified, deleted or normal.

Here, we present a method (and associated tool: ‘conliga’) that uses a fully probabilistic approach to infer relative copy number (RCN) alterations at each locus from FAST-SeqS data. conliga provides an RCN profile per sample and therefore enables this low-cost sequencing approach to be used as a SCNA assay.

## Results

### Development of probabilistic model

Based on observations of raw data (Supplementary Note 2, Supplementary Fig. S1), we created a probabilistic model (Methods, Supplementary Note 3). The model takes account of the observed bias in loci counts, which predominantly results from unequal PCR efficiencies between loci. Since neighbouring loci are likely to share the same copy number, we use a hidden Markov model (HMM) to model the spatial dependence between loci. This allows loci with high counts to share statistical strength with neighbouring loci, enabling us to infer contiguous regions of copy number more accurately. Moreover, we use a Bayesian nonparametric approach (sticky HDP-HMM) [26] to address the issue of the unknown number of copy number levels present in a given sample a priori (Methods). We use Markov chain Monte Carlo (MCMC) methods to infer the RCN of each locus, plus all other latent variables in the model (Methods, Supplementary Table S1, Supplementary Notes 4, 5 and 6). This enables us to provide the uncertainty of the RCN estimates, summarised by credible intervals, in conliga’s standard output.

### Application to high-purity oesophageal adenocarcinoma samples and performance comparison to other methods

To test our method, we analysed 11 oesophageal adenocarcinoma tumours (Methods, Supplementary Tables S2 and S3), which had been sequenced using HC WGS (>50X) and FAST-SeqS. In addition, we downsampled the WGS data of each sample to nine million reads to simulate typical LC WGS (∼0.1X coverage) samples (Methods). We compared the copy number calls derived from ASCAT [27] applied to HC WGS data, with the RCN calls from QDNAseq [22] applied to LC WGS data, CNVkit applied to FAST-SeqS data and conliga applied to FAST-SeqS data (Methods).

**Fig. 1:**
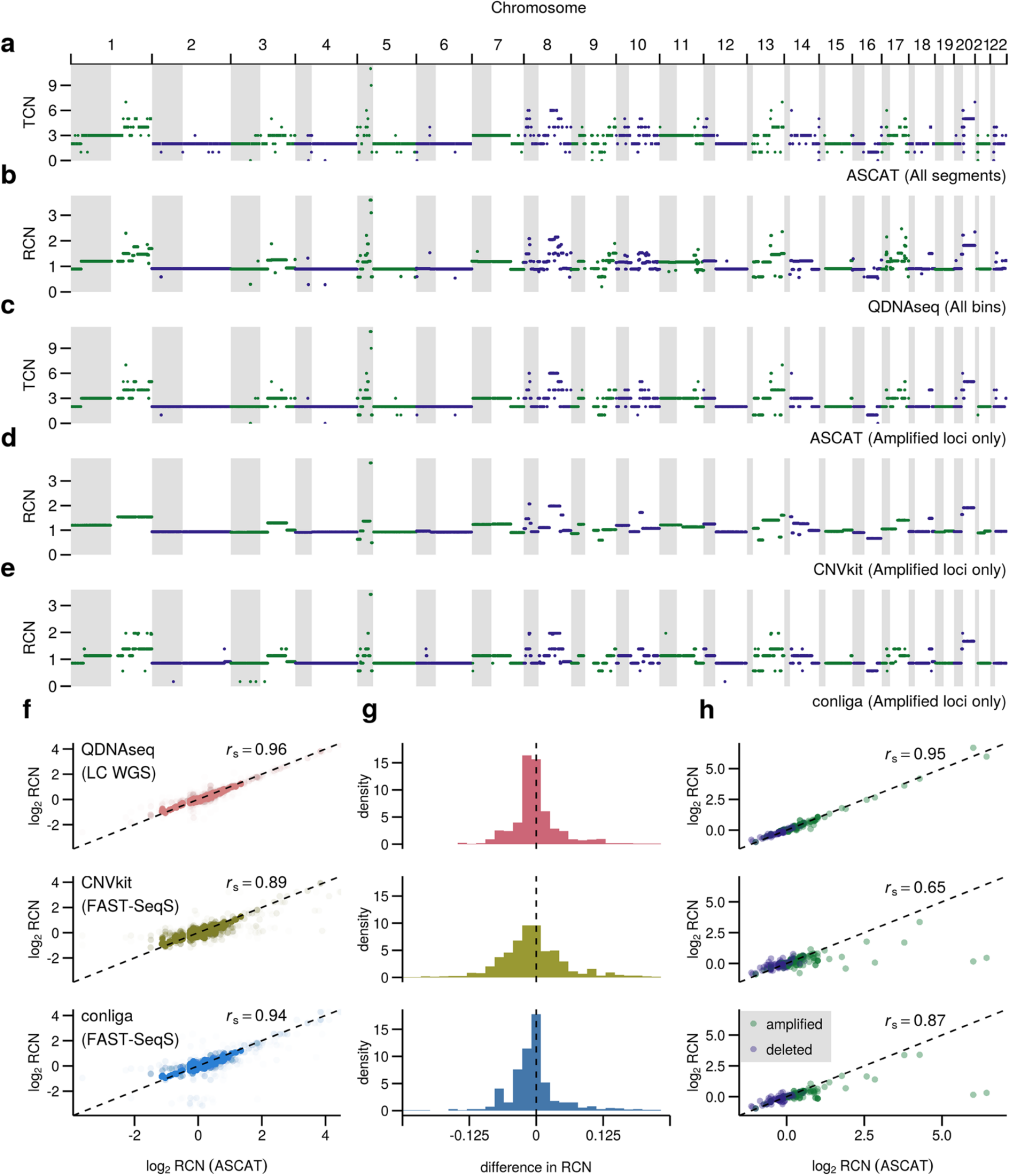
Comparison of conliga method with ASCAT, QDNAseq and CNVkit. **a** Total copy number (TCN) profile determined by ASCAT using HC WGS data for sample OAC2, showing all copy number segments. **b** RCN profile determined by QDNAseq using LC WGS data for sample OAC2, showing all 15 Kbp bins. **c** TCN profile determined by ASCAT from HC WGS data, **d** RCN profile determined by CNVkit, **e** RCN profile determined by conliga, all (**c**-**e**) showing copy number calls at the intersection of ASCAT’s called regions and FAST-SeqS loci for sample OAC2. **f** Comparison of log_2_ RCN calls from 11 samples between QDNAseq and ASCAT (top), CNVkit and ASCAT (middle) and conliga and ASCAT (bottom). *r*_*s*_ represents the Spearman’s rank correlation coefficient. All RCN calls at the intersection of ASCAT’s called regions, QDNAseq 15Kb bins and FAST-SeqS loci in all 11 OAC samples are shown as points. **g** Distribution of differences between ASCAT RCN calls and QDNAseq RCN estimates for 11 OAC samples (top), ASCAT RCN calls and CNVkit RCN estimates for 11 OAC samples (middle) and ASCAT RCN calls and conliga RCN estimates for 11 OAC samples (bottom). **h** Comparison of performance at gene-level resolution between ASCAT and QDNAseq for 36 selected genes (top), ASCAT and CNVkit (middle) and ASCAT and conliga (bottom). The values represent the weighted mean of RCN calls at each gene for each of the 11 OAC samples (Methods)

In Fig. 1a-e, we demonstrate that similar SCNA profiles are obtained with the four methods for an example sample (OAC2) and that high-resolution SCNA information is maintained by sampling genomic loci using FAST-SeqS. As is evidenced by the copy number calls on chromosomes 1, 5, 8, 10, 13 and 17 for this sample, conliga provides greater detail in its inferred RCN profile than CNVkit and is more comparable to those inferred by ASCAT and QDNAseq. Figure 1f shows the performance of conliga, CNVkit and QDNAseq across all 11 OAC samples, measured by comparing each tool’s RCN calls with those inferred by ASCAT (Methods). The Spearman’s rank correlation coefficient between ASCAT’s RCN calls (gold-standard) and conliga, CNVkit and QDNAseq was 0.94, 0.89 and 0.96, respectively. In Fig. 1g, we see that when compared with CNVkit, conliga’s RCN calls tend to be closer to those of ASCAT’s and conliga has more comparable performance to QDNAseq in this regard.

From the literature [15, 28] we selected a set of 36 genes that have been observed to be recurrently amplified or deleted in OAC (Supplementary Table S4, Methods). We determined the weighted mean of the RCN calls for these genes for each sample via each method (Methods, Supplementary Tables S5 and S6). The Spearman’s rank correlation coefficient between ASCAT’s weighted mean RCN calls (gold-standard) and conliga, CNVkit and QDNAseq was 0.87, 0.65 and 0.95, respectively. In Fig. 1g, we see that there are two instances from 396 comparisons (36 genes x 11 samples) where a substantially different result would be achieved by conliga. Even within this panel of 36, it is notable that 13 genes harbour FAST-SeqS loci (Supplementary Tables S7 and S8), providing evidence of intra gene SCNAs in some cases, such as the focal deletions detected by conliga in *FHIT*, *PARK2*, and *MACROD2* (Supplementary Fig. S2).

### In silico dilution series and limit of SCNA detection

The purity of tumour samples obtained by dissection can vary widely [29], as can samples obtained non-invasively, e.g. ctDNA from plasma [30]. As tumour purity reduces, the copy number signal-to-noise ratio decreases. To determine the performance of conliga, CNVkit and QDNAseq under different purity conditions, we generated samples with varying purity by mixing sequencing reads from normal and OAC samples (Methods). FAST-SeqS samples were generated with two million reads and LC WGS samples were generated with nine million reads.

**Fig. 2:**
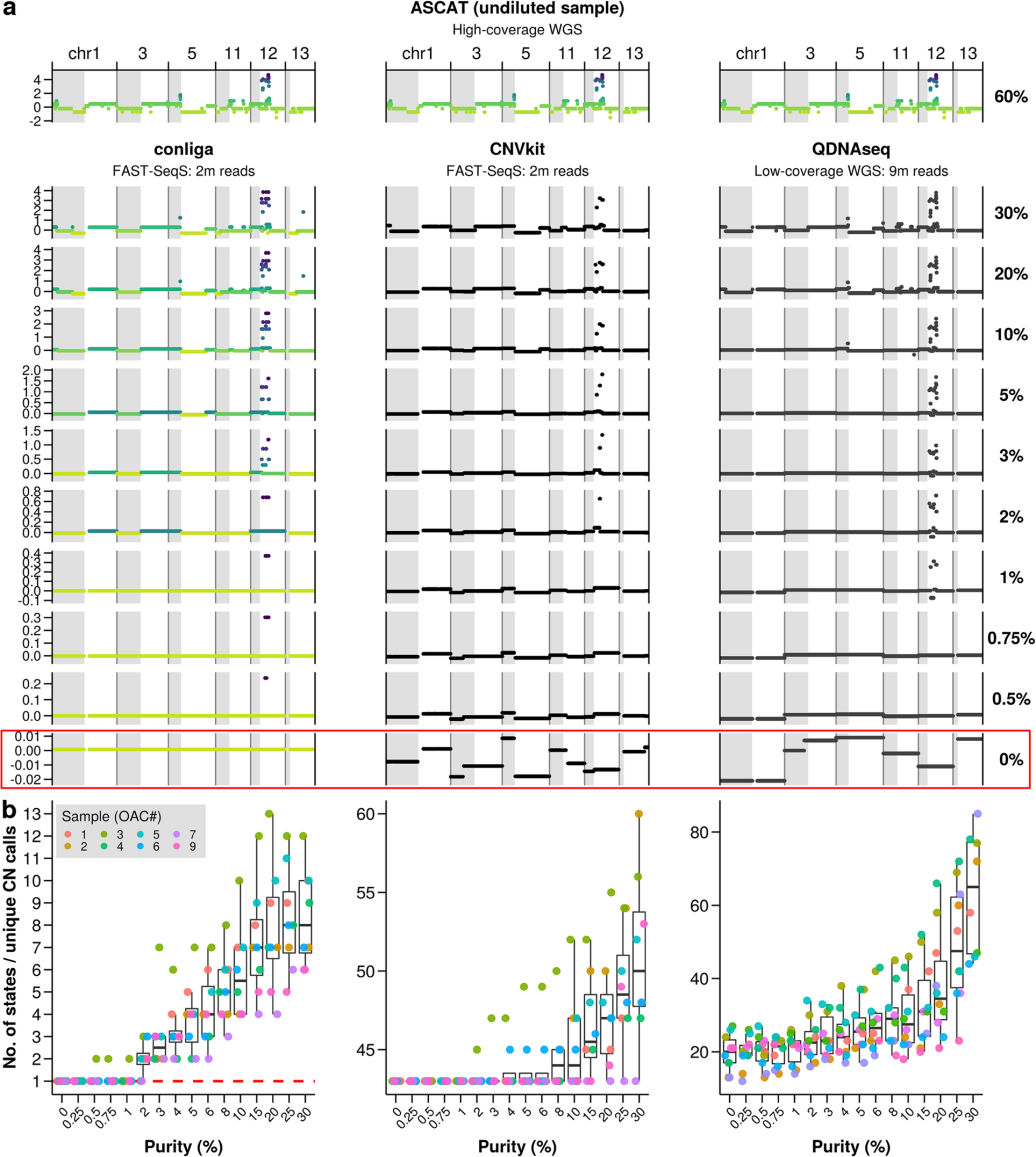
Comparing the performance of SCNA detection in low tumour purity samples and determining the limit of detection. **a** Left column: RCN calls by conliga, showing a selection of chromosomes, at different dilutions of sample OAC3, compared to the ASCAT RCN profile of the undiluted sample (top left), discrete copy number states are coloured with a gradient (light green to purple), highlighting regions with differing SCNAs. Middle column: RCN calls by CNVkit at different dilutions of sample OAC3, compared to ASCAT RCN profile (top middle). Right column: RCN calls by QDNAseq at different dilutions of sample OAC3, compared to ASCAT RCN profile (top right). Calls by CNVkit and QDNAseq are not coloured because they do not provide hidden state information. Purity levels are indicated as a percentage on the right-hand side. 0% purity profiles are highlighted in a red box, for which all regions of the genome should have equal RCN. **b** The number of copy number states detected by conliga (left) in each of eight OAC samples at differing purity levels. The limit of detection is determined by the lowest purity level in which more than one copy number state is detected. Red dashed line indicates one hidden state inferred, indicating that zero copy number changes are inferred. The number of unique RCN calls observed within each sample for CNVkit (middle) and QDNAseq (right). Note the differing y-axis ranges and that the number of unique RCN calls are always greater than one for CNVkit and QDNAseq

Figure 2a shows the inferred RCN profiles obtained for each method at varying purity levels for sample OAC3. At 30% purity, conliga, CNVkit and QDNAseq recapitulate the copy number profile of the undiluted sample as determined by ASCAT. It is noticeable that CNVkit does not capture as much detail of the profile, for example on chromosome 12. The focal amplification on chromosome 12 is identified by conliga at 0.75% and 0.5% purity and not detected by QDNAseq below 1% or CNVkit below 2%. At 5% purity, other than the focal amplification on chromosome 12, QDNAseq fails to detect subchromosomal SCNAs, whereas conliga shows evidence of chromosome arm and subchromosomal arm changes. At 2% purity, conliga is able to distinguish some of the more prominent chromosomal arm SCNAs. It appears that CNVkit detects subchromosomal SCNAs at all purity levels. However, we see a similar RCN profile inferred at 0% purity, where the expectation is that all regions of the genome have equal copy number, suggesting that these inferred differences in RCN are due to noise generated by segmentation. The same is true for QDNAseq. Indeed, it is difficult to distinguish true SCNAs from noise generated by segmentation in the RCN profiles inferred by QDNAseq and CNVkit, while this is not the case for conliga’s inferred profile.

In Fig. 2b, we show that conliga is able to detect SCNAs at 3% purity in all samples (eight), five at 2% and one at 0.5%. Here, the limit of detection is determined by the number of hidden states inferred for each sample; one hidden state means SCNAs were not detected and more than one hidden state means SCNAs were detected. With conliga, long chromosomal arm amplifications can be detected at 2-3% purity, while some focal amplifications (particularly those occurring at loci with a bias towards obtaining a high number of counts) can be detected at <1% purity (e.g. chr12 in OAC3, Fig. 2a). CNVkit and QDNAseq do not provide hidden state information, so we used the number of unique RCN calls inferred for each sample instead. Even at 0% purity, we see that there are substantially more than one unique RCN call per sample. This is the result of regions of the genome being segmented independently, resulting in segmentation error, which obscures the biological signal and makes it difficult to determine the limit of detection for CNVkit and QDNAseq. CNVkit segments each chromosome arm independently [20], with each 0% purity sample having inferred 43 unique RCN calls. Using 43 RCN calls as the baseline, we see that CNVkit detects SCNAs in two out of eight samples at 4-6% purity, four out of eight samples at 10%, and fails to detect SCNAs for any purity level of OAC7 (up to 30% purity level tested).

### Detection of clinically-relevant SCNAs in pre-malignant disease and patient cohorts

SCNA load rather than SNVs within driver genes distinguishes OAC from its associated pre-malignant lesion, Barrett’s oesophagus (BO). To assess whether we were able to detect SCNAs in pre-malignant samples, we processed BO samples with various grades of dysplasia using FAST-SeqS and inferred their associated RCN profiles using conliga. We were able to detect clinically-relevant copy number alterations, such as evidence for focal gains of *PRKCI*, *ERBB2* and *GATA6* and deletions of regions containing *CDKN2A*, *PTPRD*, *SMAD4* and *TP53* (Supplementary Fig. S2).

In addition to use as a detection tool, inexpensive production of FAST-SeqS data allows for large cohorts of patients to be studied to find relationships between SCNA profiles and response to therapies, for example. With this in mind, we looked at the average SCNA profiles across small cohorts of patients with OAC, gastric adenocarcinoma (GAC) and BO (Supplementary Fig. S2, Methods), which highlighted amplifications of known oncogenes such as *EGFR*, *MYC*, *GATA4*, and *MDM2*, some with known drug targets, and deletions of tumour suppressor genes, e.g. *FHIT*, *TP53*, *SMAD4* and *RUNX1*.

## Discussion

Work by Ciriello et al. [3] suggests that either somatic single-nucleotide variants (SNVs) or SCNAs can drive oncogenesis. However, there is a bias towards screening for SNVs using targeted gene panels [31] meaning SCNA-driven cancers, such as OAC, may not be detected. Our data suggest that there is potential for FAST-SeqS and conliga to be used alongside existing low-cost gene panels to detect SCNAs, in addition to SNVs, to screen and surveil patients for the development of cancer. Other potential uses include low-cost screening of samples in large-scale cancer genomes studies, such as the International Cancer Genome Consortium (ICGC) [32] and The Cancer Genome Atlas (TCGA) [33] projects, prior to further genomic analyses. Furthermore, due to the low-cost and low-input DNA required, several spatially or temporally related samples can be analysed for the purposes of determining how SCNAs accumulate in normal tissues and contribute to tumour evolution, similar to previous studies on somatic mutations in the eyelid epidermis [34].

We showed that conliga (applied to FAST-SeqS data) had better performance in inferring RCN profiles from high-purity cancer samples than CNVkit (also applied to FAST-SeqS data), and a lower, though comparable performance to QDNAseq (applied to LC WGS data). It should be noted that this analysis is biased in favour of QDNAseq. To obtain the LC WGS reads used by QDNAseq, we downsampled the reads from the same HC WGS sample that was used for ASCAT, which was used as the gold-standard comparison. A fairer comparison would have been to perform separate library preparation and sequencing for the samples processed by QDNAseq. By not doing this, we may have inflated QDNAseq’s performance. Furthermore, we used nine million reads to obtain 0.1X coverage, which is commonly used for LC WGS copy number profiling [22]. The corresponding FAST-SeqS samples had fewer reads (min: 607,073, mean: 1,806,904, max: 4,651,028). If we were to increase the number of FAST-SeqS reads to nine million, we would likely see an increase in conliga’s performance.

Another limitation of the comparison to ASCAT is that ASCAT’s assumption of a fully clonal sample places a restriction on the copy number profiles it can infer, which is not an assumption or restriction shared by QDNAseq, CNVkit and conliga. This will be one source of the RCN differences observed between ASCAT and the other methods. However, since this limitation applies equally to each tested method, it does not bias the results in favour of one method over the other.

CNVkit and QDNAseq both use a popular change point detection algorithm, circular binary segmentation [35], and apply this to regions of the genome independently [20, 22]. This means that genomic regions with the same copy number may be assigned different RCN values, which we refer to as segmentation error. As we showed in Fig. 2b, this error introduced by segmentation may obscure the RCN signal in the data, making it difficult to distinguish between true copy number differences between regions of the genome and segmentation error. This may not be problematic when profiling high-purity samples, since the RCN signal tends to be substantially greater than the error introduced. However, in cancer screening and surveillance, samples are often obtained non-invasively and, either 1) they are of low tumour purity if tumour DNA is present or 2) they will contain no tumour DNA if the sample is normal. Discriminating between these two cases is critical in cancer detection and surveillance. For low-purity samples, regions with differing copy number will have relatively small RCN differences typically, and discriminating between genuine copy number changes and segmentation error is more challenging. An advantage of conliga over other methods is its use of HMMs and its Bayesian nonparametric approach, specifically its use of the Sticky HDP-HMM [26]. This allows the total number of distinct copy numbers in the sample to be inferred from the data. As we showed in the in silico dilution experiment, this gives us a natural way to distinguish between samples with and without inferred SCNAs.

The Dirichlet process prior, that is part of the conliga model, biases the model against creating additional copy number states when there is little evidence to do so. One disadvantage is that this means that copy numbers that involve few loci, for example focal amplifications with differing copy numbers, may be merged into one copy number state. We can see the effect of this in Fig. 1, when comparing the profiles inferred by ASCAT and conliga; regions that are inferred to be copy number 5 and 6 by ASCAT are inferred to have the same copy number by conliga. This is also the case for regions inferred to be copy number 9 and 11 by ASCAT. However, the ability to discern a difference in copy number between regions that are highly amplified may have limited clinical utility in practice. As such, we believe the advantages of using of the Dirichlet process prior outweigh the disadvantages.

As we saw in Fig. 2a and b, the number of copy number states inferred by conliga decrease as tumour purity reduces. Assuming two million reads per sample and high tumour purity (>60%), aside from high copy numbers with few affected loci, we would expect conliga to accurately detect and quantify most copy numbers present in a sample. In the high-purity case, we would expect that most inferred copy number states should provide a one-to-one mapping to true underlying copy number signals in the data, as we saw in Fig. 1e for example. As tumour purity reduces below 30%, we would expect copy number signals in the data to be increasingly merged together into the same copy number state, as we observed in Fig. 2. Our results suggest that conliga can detect some chromosome-arm level alterations between 2-5% tumour purity, but that we cannot expect conliga to detect these large alterations below 2% without increasing the number of reads per sample. As we discuss below, under certain conditions, conliga may be able to detect highly focal amplifications below 1%.

By modelling the loci counts with an appropriate parametric count distribution (beta-binomial) and the spatial dependence between loci with a HMM, loci that tend to receive greater read counts can propagate their increased statistical strength regionally to neighbouring loci and globally to their associated hidden states to infer more accurate RCN profiles. We believe this is one of the reasons why conliga has better performance than CNVkit in inferring RCN profiles from FAST-SeqS data. There are substantial differences between the PCR efficiencies of FAST-SeqS loci and, as a consequence, large differences in the magnitude of read counts between loci (Supplementary Fig. S1). The loci with higher counts carry greater influence than the noisier, lower count loci on the inference of RCN profiles, due to the model implemented.

In Fig. 1h and Supplementary Fig. S2, we observed that conliga was able to detect amplifications of known oncogenes, some with known drugs targets, and deletions of tumour suppressor genes in OAC, BO and GAC samples. Of the 36 frequently amplified and deleted genes in OAC, 13 were found to contain FAST-SeqS loci within their gene boundaries. This meant that in some cases, conliga could identify intra-gene deletions within tumour suppressor genes. Focal deletions such as these may be functionally relevant, potentially rendering tumour suppressor genes inactive. The ability of conliga to detect focal alterations depends on the characteristics of the associated genomic region (in particular, e.g., the effect size, the number of loci affected, and their associated amplification biases etc). As we saw in Fig. 2, conliga was able to detect a focal amplification in chromosome 12 at a lower tumour purity than QDNAseq and CNVkit. Indeed, this region contained loci that tend to receive high read counts, which helped to detect the alteration at purities as low as 0.5%. It is worth noting that, due to their larger effect sizes, it is easier to detect highly amplified regions in low purity samples than to detect deletions, which have smaller effect sizes. We would not expect conliga to detect focal deletions with regularity, particularly if tumour purity is low or if the deleted region does not contain FAST-SeqS loci that tend to have high counts (high amplification bias).

FAST-SeqS would not be the assay of choice if one is only interested in a small gene panel. However, there are only two instances from 396 comparisons (36 genes x 11 samples) where a substantially different result would be achieved by conliga compared with ASCAT (Fig. 1h). In these two instances, we saw that conliga and CNVkit did not detect very highly focal amplifications of recurrently amplified genes. On inspection of the ASCAT results, these two amplified regions were so narrow as to fall between two FAST-SeqS loci. Naturally, conliga and CNVkit would be able to detect these alterations if there are no FAST-SeqS loci located in the altered regions. Furthermore, the single primer pair used in FAST-SeqS amplifies primate-specific LINE1s dispersed throughout the genome. Indeed, it has been shown that genomic deletions can occur due to unequal homologous recombination between two repeat elements. This can lead to deletions occurring between neighbouring LINE1 elements [36, 37] and as such, FAST-SeqS would not be able to provide evidence of such deletions. These are limitations of the FAST-SeqS assay, and users should bear this in mind before use. As we show in Supplementary Fig. S1, the distribution of distances between neighbouring loci has a heavy right tail. While the median distance is approximately 120 Kbp (after filtering for robust loci), there are some regions of the genome that have larger gaps between FAST-SeqS loci that are greater than 1 Mbp in length. One avenue of further work could be to extend conliga to infer SCNA profiles using targeted gene panel data jointly with FAST-SeqS data. In doing so, we could avoid missing SCNAs that involve important and clinically relevant genes.

Another approach to reduce the chance of missing regions of interest would be to use RealSeqS [38] instead of FAST-SeqS. RealSeqS uses a single-primer pair to amplify a greater number (∼350,000) of repetitive elements dispersed throughout the genome [38]. The associated machine learning method used to analyse RealSeqS data aims to provide an overall aneuploidy score for each sample. Subchromosomal arm features are used as input to determine this score. However, these features are limited to contiguous regions of at least 5 Mb in length [38]. We would expect the properties of FAST-SeqS and RealSeqS data to be similar, and potentially conliga could be applied to RealSeqS data to provide an amplicon-level copy number profile. Further work would be required to determine if conliga can be applied to RealSeqS data, or whether alterations to the method and algorithms would be required. If it can be applied, a higher-resolution copy number profile could be obtained compared with those obtained using FAST-SeqS data. However, increased sequencing per sample would be required in order to obtain a similar sequencing depth to FAST-SeqS data, which would increase costs. Avenues for future study include applying conliga to RealSeqS data to determine the benefit of the increased spatial resolution and determining the sequencing depth required to achieve similar or improved performance compared with conliga applied to FAST-SeqS. Furthermore, we hypothesise that greater sensitivity could be achieved if amplicon-level copy number profiles are used as features for cancer detection, compared with current approaches that use less granular features to determine sample-level aneuploidy scores. More generally, we envisage further development of repetitive element sequencing to reduce technical variability and modifications of the assays to alter the number of reads obtained at specific loci to increase statistical power in regions of interest.

## Conclusions

We have shown that FAST-SeqS data can be used as a viable, inexpensive, and simple alternative to LC WGS for the purpose of SCNA detection and quantification. conliga provides accurate and high-resolution SCNA profiles across the genome and at regions of interest such as oncogenes and tumour suppressors. conliga (applied to FAST-SeqS data with two million reads per sample) is particularly useful for detecting and discriminating SCNAs in low purity samples. We believe that conliga makes FAST-SeqS a more clinically valuable diagnostic assay to detect and monitor patients for the development of cancer, as well as a useful research tool, enabling inexpensive and fast SCNA profiling of cancer samples.

## Methods

### conliga: statistical model

#### Statistical model for sample counts

We model the sample counts, in *L* selected loci, by assuming that the count at locus *l* in chromosome arm *r* in sample *j* is distributed:

1 $$  y_{r,l,j} \sim \text{Binomial}(n_{j}, \theta_{r,l,j})  $$

Here, *n*_*j*_ is the total number of sequencing reads aligned to the *L* loci in sample *j*, *θ*_*r*,*l*,*j*_ represents the probability of observing an aligned read at locus *l* in chromosome arm *r* in sample *j*. We model *θ*_*r*,*l*,*j*_ as follows:

2 $$  \theta_{r,l,j} \sim \text{Beta}(s_{j} \hat{c}_{r,l,j} m_{r,l}, s_{j} (1 - \hat{c}_{r,l,j} m_{r,l}))  $$

Here, *s*_*j*_ is the inverse dispersion variable for sample *j* where *s*_*j*_>0, *m*_*r*,*l*_ represents the probability of an aligned sequencing read originating from locus *l* in chromosome arm *r* in a control sample, where $\sum _{r} \sum _{l=1}^{L_{r}} m_{r,l} = 1$ and $\hat {c}_{r,l,j}$ is the relative copy number at locus *l* in chromosome arm *r* in sample *j*. The number of loci in each chromosome arm is denoted as *L*_*r*_ and so the total number of loci, $L = \sum _{r} L_{r}$.

We can interpret ***m*** as defining the bias in observing aligned read counts from the FAST-SeqS protocol. This bias can be explained by unequal PCR efficiencies between loci in addition to biases in aligning reads uniquely to FAST-SeqS loci, among other factors. Note that:

3 $$  \mathbb{E}\left[{\theta_{r,l,j}}\right] = \hat{c}_{r,l,j} m_{r,l}  $$

We can interpret this equation intuitively; the relative copy number scales the probability of reads to align to a locus. For example, if the relative copy number of a locus is 2 we expect the proportion of reads at the locus to double. This fits with our observations shown in Supplementary Fig. S1.

The inverse dispersion variable, *s*_*j*_, is sample specific and reflects our observations that the level of dispersion varies between samples. This variation in dispersion between samples might be due to varying levels of DNA degradation and/or varying quantities of starting material between samples, among other factors. *s*_*j*_ relates to the variance and the mean of *θ*_*r*,*l*,*j*_ in the following way:

4 $$  \text{Var}\left(\theta_{r,l,j} \right) = \frac{1}{s_{j} + 1} \left(\mathbb{E}\left[{\theta_{r,l,j}}\right] - \mathbb{E}\left[{\theta_{r,l,j}}\right]^{2} \right)  $$

The expected count, *y*_*r*,*l*,*j*_, in chromosome arm *r* at locus *l* in sample *j* is:

5 $$  \mathbb{E}\left[{y_{r,l,j} \mid \theta_{r,l,j}}\right] = \mu = n_{j} \hat{c}_{r,l,j} m_{r,l}  $$

The variance of *y*_*r*,*l*,*j*_ can be written as a quadratic function of *μ* with the coefficients being a function of *n*_*j*_ and *s*_*j*_:

6 $$  {}\text{Var}\left(y_{r,l,j} \mid \theta_{r,l,j} \right) = \left(1 + \frac{n_{j}-1}{s_{j}+1} \right) \mu - \left(\frac{1}{n_{j}} + \frac{n_{j}-1}{s_{j}+1} \right) \mu^{2}  $$

Note that in the limit *s*_*j*_→*∞*, a binomial noise model is recovered.

#### Probabilistic generative model of loci counts for control samples

We assume that the loci within a control sample, *k*, have equal copy numbers (diploid). This means that the RCN for each locus is 1. By setting $\hat {c}_{r,l,k} = 1$, we model the generative process of counts from a control sample as follows:

7 $$  \begin{aligned} s_{k} \mid \psi & \sim \text{Gamma}(\psi_{\text{shape}}, \psi_{\text{scale}})\\ m_{r,l} \mid \phi & \sim \text{Beta}(\phi_{c,r,l}, \phi_{d,r,l})\\ \theta_{r,l,k} \mid s_{k}, m_{r,l} & \sim \text{Beta}(s_{k} m_{r,l}, s_{k} (1 - m_{r,l}))\\ x_{r,l,k} \mid \theta_{r,l,k}, n_{k} & \sim \text{Binomial}(n_{k}, \theta_{r,l,k}) \end{aligned}  $$

Here, Gamma(*ψ*_*shape*_,*ψ*_*scale*_) represents the prior distribution over the sample specific inverse dispersion parameter, *s*_*k*_, and Beta(*ϕ*_*c*,*r*,*l*_,*ϕ*_*d*,*r*,*l*_) defines the prior distribution over *m*_*r*,*l*_.

#### Linking FAST-SeqS loci using a hidden Markov model

We assume that chromosome arms are independent. By that we mean, the RCN of the first locus in arm q is independent of the RCN of the last locus in arm p from the same chromosome (and all other chromosome arms). As such, we model each chromosome arm as an independent Markov chain for each sample *j*. We denote (note that for simplicity we have dropped the sample index *j*):

- *z*_*r*,*l*_ as the *hidden state* (or *copy number state*) of the Markov chain at locus *l* in chromosome arm *r*
- *π*^0^ as the *initial distribution* of the first locus (*l*=1), in chromosome *r*
- *π*_*u*_ as the *transition distribution* for hidden state, *u*
- $\hat {c}_{u}$ as the *relative copy number* associated with hidden state, *u*.

The first locus of a chromosome arm (*l*=1) is distributed:

8 $$ z_{r,1} \sim \pi^{0}  $$

For all other loci (*l*>1):

9 $$ z_{r,l} \mid z_{r,l-1} \sim \pi_{(z_{r,l-1})}  $$

The count, *y*_*r*,*l*_, at locus *l* in chromosome arm *r* is conditionally independent of the hidden states and observations of other loci:

10 $$ {}\begin{aligned} \theta_{r,l} \mid \hat{\boldsymbol{c}}, z_{r,l}, m_{r,l}, s & \sim \text{Beta}(s \hat{c}_{z_{r,l}} m_{r,l}, s (1 - \hat{c}_{z_{r,l}} m_{r,l}))\\ y_{r,l} \mid \theta_{r,l}, n & \sim \text{Binomial}(n, \theta_{r,l}) \end{aligned}  $$

The joint density for *L*_*r*_ loci in chromosome arm *r* is:

11 $$ \begin{aligned} &p(z_{r, 1:L_{r}},y_{r, 1:L_{r}}, \theta_{r, 1:L_{r}})\\ &= p(y_{r,1} \mid z_{r,1}, \theta_{r,1}) p(\theta_{r,1} \mid z_{r,1}) p(z_{r,1})\\ &\quad \prod_{l=2}^{L_{r}} p(y_{r,l} \mid z_{r,l}, \theta_{r,l}) p(\theta_{r,l} \mid z_{r,l}) p(z_{r,l} \mid z_{r, l-1})\\ &= \pi^{0}_{z_{r,1}} p(y_{r,1} \mid z_{r,1}, \theta_{r,1}) p(\theta_{r,1} \mid z_{r,1})\\ &\quad \prod_{l=2}^{L_{r}} \pi_{z_{r,l-1}, z_{r,l}} p(y_{r,l} \mid z_{r,l}, \theta_{r,l}) p(\theta_{r,l} \mid z_{r,l}) \end{aligned}  $$

where, $z_{r,1:L_{r}}$ denotes the sequence $\{ z_{r,1}, \dots, z_{r,L_{r}} \}$, $y_{r,1:L_{r}}$ denotes $\{ y_{r,1}, \dots, y_{r,L_{r}} \}$, and $\theta _{r,1:L_{r}}$ denotes $\{ \theta _{r,1}, \dots, \theta _{r,L_{r}} \}$. The joint density for all *L* loci in the genome is given by:

12 $$ p(\boldsymbol{z}, \boldsymbol{y}, \boldsymbol{\theta}) = \prod_{r} p(z_{r, 1:L_{r}},y_{r, 1:L_{r}}, \theta_{r, 1:L_{r}})  $$

#### Probabilistic generative model of a sample’s relative copy number profile

The number of copy number states present in a sample is unknown a priori. In samples that have equal copies of each locus, only one copy number state is present. Conversely, it is possible (although unlikely) that each locus has its own unique copy number, meaning that there could be up to *L* copy number states in a sample. Additionally, we expect neighbouring loci to share the same copy number given their genomic distance from each other (Supplementary Fig. S1). To address these two features of the data, we used the sticky hierarchical Dirichlet process hidden Markov model (sticky HDP-HMM) [26] as a framework to model the generative process of a sample’s relative copy number profile. By doing so, we adequately model the spatial persistence of copy number states and allow for countably infinite numbers of states within a sample. The generative model is as follows:

13 $$  {}\begin{aligned} \beta \mid \gamma & \sim \text{GEM}(\gamma)\\ \pi^{0} \mid \alpha, \beta & \sim \text{DP}\left(\alpha, \beta \right)\\ \pi_{u} \mid \alpha, \kappa, \beta & \sim \text{DP}\left(\alpha + \kappa, \frac{\alpha \beta + \kappa \delta_{u}}{\alpha + \kappa}\right)\\ \hat{c}_{u} \mid H, \lambda & \sim H(\lambda) \\ z_{r,1} \mid \pi^{0} & \sim \pi^{0} \\ z_{r,l} \mid \{ \pi_{u} \}_{u=1}^{\infty}, z_{r,l-1} & \sim \pi_{z_{r,l-1}}, \text{ for} l > 1 \\ \tilde{s} \mid \omega & \sim \text{Gamma}(\omega_{shape}, \omega_{scale})\\ \tilde{\theta}_{r,l} \mid \{ \hat{c}_{u}\}_{u=1}^{\infty}, z_{r,l}, \hat{m}_{r,l}, \tilde{s} & \sim \text{Beta}(\tilde{s} \hat{c}_{z_{r,l}} \hat{m}_{r,l}, \tilde{s} (1 - \hat{c}_{z_{r,l}} \hat{m}_{r,l}))\\ y_{r,l} \mid \tilde{\theta}_{r,l}, \tilde{n}, & \sim \text{Binomial}(\tilde{n}, \tilde{\theta}_{r,l}) \end{aligned}  $$

Note that we use $\tilde {n}$, $\tilde {s}$, $\tilde {\theta }_{r,l}$ to distinguish these variables from those in the probabilistic model of control counts (Eq. 7) and denote them as specific to the sample with copy number profile. Here, GEM denotes the stick-breaking construction of the Dirichlet process as described in Fox et al. [26]. *γ* is a hyperparameter of the sticky HDP-HMM and represents our prior on the number of copy number states in the sample; the greater the value of *γ*, the greater number of copy number states we expect in the sample. Each row of the transition matrix, *π*_*u*_, is drawn from a Dirichlet process and depends on *β*, *α* and *κ*. It can be shown that:

14 $$ \mathbb{E}\left[{\pi_{u,v} \mid \alpha, \beta, \kappa}\right] = \frac{\alpha \beta_{v} + \kappa \delta_{u,v}}{\alpha + \kappa}  $$

where *δ*_*u*,*v*_ represents the discrete Kronecker delta function. If we define $\rho = \frac {\kappa }{\alpha + \kappa }$ (as in Fox et al. [26]) and by noting that *α*=(1−*ρ*)(*α*+*κ*), we obtain:

15 $$ \mathbb{E}\left[{\pi_{u,v} \mid \beta, \rho}\right] = (1 - \rho) \beta_{v} + \rho \delta_{u,v}  $$

As such, we see that *ρ* defines how much weight is placed on self-transition within a copy number state. The vector, *β*, itself drawn from a Dirichlet process, represents the global transition distribution and holds information about the proportion of loci expected in each copy number state.

The variance of the transition probability from copy number state *u* to *v* is given by:

16 $$ {}\text{Var}(\pi_{u,v} \mid \alpha, \beta, \kappa) \!= \! \frac{\mathbb{E}\left[{\pi_{u,v} \mid \alpha, \beta, \kappa}\right] \left(1 - \mathbb{E}\left[{\pi_{u,v} \mid \alpha, \beta, \kappa}\right] \right)}{\alpha + \kappa + 1}  $$

We see that *α*+*κ* is inversely proportional to the variance of the state transition probabilities.

*H* is the prior base distribution of the Dirichlet process and represents a parametric distribution, which in this case is a Gamma distribution, with parameters *λ*. It can be viewed as our prior probability distribution on the relative copy number values of the hidden states.

Note that $\hat {m}_{r,l}$ refers to the maximum a posteriori (MAP) value of *m*_*r*,*l*_ and is such assumed to be a known quantity in Eq. 13. For simplicity, the hyperparameters (*α*, *κ*, *γ*, *λ*, *ω* and *n*) are shown as fixed quantities in the model. In practice, *γ*, *λ*, *ω* and *n* are treated as fixed, while the model is parameterised in terms of *ρ* and (*α*+*κ*), with a Beta prior placed on *ρ* and a Gamma prior placed on (*α*+*κ*) as in Fox et al. [26]. See the section on inference for further details of prior distributions used and Supplementary Note 3 for further discussion on the model.

### Inference

#### Inference of loci count proportion bias (***m***)

Given a set of *K* control samples, and their loci counts, ***x***_***k***_, we used our model defined in Eq. 7 and Markov Chain Monte Carlo (MCMC) methods to infer the latent variables ***m*** and ***s*** (the vector of sample specific inverse dispersion parameters). A Metropolis-Hastings MCMC algorithm was used to obtain a sample of the posterior probability of *m*_*r*,*l*_ for all r and l, and *s*_*k*_, for each sample *k*. Full details of the algorithms are provided in Supplementary Notes 4 and 5. Count data for samples analysed in this study, processed by the pipeline described, are provided in Supplementary Table S9.

For each sequencing experiment, a suitable set of controls samples were used (see Supplementary Table S10 for the list of samples used in each experiment). As described in Eq. 7, control samples were assumed to have a relative copy number of one at each locus. In all experiments described in this paper, we used the following values for the hyperparameters:

- *ψ*_*shape*_=1.5, *ψ*_*scale*_=10^6^; where *ψ*_*shape*_ and *ψ*_*scale*_ define the shape and scale of the Gamma prior distribution on *s*_*k*_, respectively.
- *ϕ*_*c*,*r*,*l*_=1 and *ϕ*_*d*,*r*,*l*_=1 for all *r* and *l*; i.e. we used a flat Beta(1,1) prior for all *m*_*r*,*l*_

In each sequencing experiment, 20,000 iterations of the MCMC were run and the first 5,000 iterations were discarded (burn-in). Maximum a posteriori (MAP) estimates of ***m*** (denoted as $\hat {\boldsymbol {m}}$) were obtained by determining the mode of the sampled posterior densities for each locus using the KernSmooth R package [39]. Note that the MAP estimates are unlikely to sum to one exactly, as such we rescale them so that they sum to 1.

#### Inference of relative copy number profile

Given $\hat {\boldsymbol {m}}$ and the loci counts (***y***) for a sample with unknown copy number profile, we used the generative model defined in Eq. 13 and MCMC methods (based on algorithm 3 in Fox et al. [26]) to infer the latent variables in our model. MCMC methods were used to obtain a sample of the posterior probability of the hidden state of each locus (*z*_*r*,*l*_ for all *r* and *l*), the relative copy number of each hidden state ($\hat {c}_{u}$), the sample specific inverse dispersion ($\tilde {s}$), along with other latent variables in our generative model. Full details of the MCMC algorithms can be found in Supplementary Notes 4 and 6. In all experiments described in this paper, we used the following values for the hyperparameters:

- *γ*=1
- Gamma(2000,10) prior distribution (defined by shape and scale) was placed on (*α*+*κ*)
- Beta(100000,100) prior was placed on *ρ*
- Gamma(3,1) prior distribution (defined by shape and scale) was placed on the relative copy number value of the hidden states; the shape and scale parameters are defined by *λ* in Eq. 13
- *ω*_*shape*_=1.5, *ω*_*scale*_=10^6^; where *ω*_*shape*_ and *ω*_*scale*_ define the shape and scale of the Gamma prior distribution on $\tilde {s}$, respectively

The output of the MCMC was summarised in two main ways, 1) by marginalizing out the copy number state information and computing the MAP estimate (using KernSmooth R package [39]) and credible interval of the relative copy number of each locus, 2) by making use of the copy number state assignments in the following way:

1. We determined the MAP number of states observed in the MCMC chain (after burn-in). This was achieved by calculating the number of populated states in each iteration of the MCMC, and then choosing the most frequently observed number of populated states. Note that a state was considered populated in an iteration of the MCMC if at least one locus was assigned to it.
2. We filtered the iterations of the MCMC (after burn-in), choosing only those iterations that had the number of populated states equal to the MAP number of states.
3. We used the Stephens algorithm (algorithm 2 in the paper) [40] along with the Hungarian (Munkres) algorithm [41] to relabel the states, to resolve the label switching problem inherent in MCMC methods.
4. We calculated the MAP estimate and credible intervals for the relative copy number values of each relabelled state.
5. We assigned each locus to a relabelled state, choosing the relabelled state it was most frequently assigned to in the filtered iterations of the MCMC chain.

For the results presented in Fig. 2, summarisation method 2 was used. For all other results presented in the paper, summarisation method 1 was used. For the oesophageal cancer, gastric cancer and Barrett’s oesophagus samples, 50,000 iterations of the MCMC were run and the chain was thinned such that every 5th iteration of the MCMC was output to file. Additionally, the first 20,000 iterations of the MCMC were discarded (burn-in), to ensure the Markov chain had reached its equilibrium distribution. For the in silico diluted samples, presented in Fig. 2, 30,000 iterations were run, with the chain thinned so that every 5th sample was output to file and the first 5,000 iterations of the MCMC were discarded.

### Sample preparation and sequencing of samples

#### Source of samples

All samples were obtained with written informed consent. OAC, blood and GAC samples were obtained under Oesophageal Cancer Clinical and Molecular Stratification (OCCAMS) (Ethics number 10/H0305/1). OAC and GAC were obtained either as an endoscopic sample or from surgical resection specimens and stored as fresh frozen tissue. Barrett’s oesophagus tissue was obtained under BEST2: East of England–Cambridge Central Research Ethics Committee (No: 10/H0308/71). Samples of dysplastic Barrett’s oesophagus were obtained from patients with a prior diagnosis of Barrett’s oesophagus attending for their routine surveillance endoscopy. Barrett’s oesophagus was defined as the presence of ≥1 cm of columnar lined oesophagus continuous with the squamocolumnar junction.

All tissue underwent expert histopathological assessment using a haematoxylin and eosin stain from a section adjacent to the sections from which DNA was extracted. All specimen were reviewed by two expert histopathologists. Cellularity estimates were performed by two expert histopathologists and, where there was a major discordance, resolved by a third expert histopathologist.

#### Sample preparation and generation of FAST-SeqS data

Sequencing libraries were prepared using two rounds of PCR, using a similar protocol to previously published methods [23, 24]. Each extracted DNA sample underwent a 50 *μ*l first round PCR reaction with 10 *μ*l 5x Phusion HF Buffer (ThermoFisher Scientific), 1 *μ*l 10 mM dNTP (ThermoFisher Scientific), 5 *μ*l of both the forward and reverse primers (0.5 *μ*M) each (Sigma-Aldrich), 0.5 *μ*l Phusion Hot Start II DNA Polymerase 2U/ *μ*l, 5-10 *μ*l DNA template depending on the extracted concentration, and RNAse free water to make the total reaction volume. The cycling conditions for the L1PA7 primers were 98 ^∘^C for 120 s followed 2 cycles of 98 ^∘^C for 10 s, 57 ^∘^C for 120 s, and 72 ^∘^C for 120 s. The second round was also carried out as a 50 *μ*l sample reaction using 20 *μ*l taken from the first round. The rest of the reaction constituents were the same as the first round reaction, with the exception of primers (Supplementary Table S11), which contained a unique index for each sample. The cycling conditions for the second round reaction were 98 ^∘^C for 120 s followed by 13 cycles of 98 ^∘^C for 10 s, 65 ^∘^C for 15 s, and 72 ^∘^C for 15 s for all the primers. After the second round, samples underwent quantification using the 2200 TapeStation (Agilent), Agilent 2100 Bioanalyser (Agilent) and Kapa quantification (KapaBiosystems) prior to submission for sequencing. The samples were then pooled in equimolar concentrations and gel extracted according to manufacturer’s instructions (Qiaquick Gel Extraction Kit, Qiagen). Finally, the samples were submitted for sequencing on a MiSeq (Illumina) platform. All samples were run with 20% PhiX to increase complexity for sequencing. Sequencing was performed as 150bp single end. Samples were run with at least three normal controls prepared at the same time and sequenced on the same platform.

#### Sample preparation and generation of high-coverage WGS data

WGS library preparation and sequencing was performed as previously described by Secrier et al. [9].

#### In silico generation of low-coverage WGS data

For our purposes, LC WGS data were defined as nine million single-end 50 base pair reads per sample because this was the type of data analysed in Scheinin et al. [22]. Samples are typically multiplexed together and sequenced on a single Illumina sequencing lane. After processing and alignment of the reads, we expect approximately 0.1X coverage of the genome (as per analysis described in Scheinin et al.). We obtained LC WGS data by downsampling reads from HC WGS BAM files in the following way:

1. We selected a subset of the alignments, containing only reads sequenced on a single lane (chosen to be the lane from the first read in the BAM file), and trimmed the reads and Phred scores to the first 50 base pairs using a custom Bash script.
2. The resulting alignments were filtered (using samtools [42] version 0.1.18), excluding those that were secondary alignments (-F 256) and including only those that were first in a pair (-f 64) and output to a new BAM file.
3. This BAM file was downsampled to 9 million reads/alignments using the DownsampleSam command from Picard tools (http://broadinstitute.github.io/picard, version 2.9.1) using the "Chained" strategy.
4. The resulting BAM file was converted to FASTQ by SamToFastq (Picard tools).
5. The FASTQ file was aligned to GRCh38 (GenBank accession: GCA_000001405.15, no alt analysis set) using BWA-backtrack (bwa samse and bwa aln, version 0.7.15-r1140) [43], which is more suitable for reads below 70 base pairs in length.
6. In the resulting BAM file, we removed PCR duplicates and removed alignments with mapping quality below 37 as per the analysis undertaken by Scheinin et al. [22] using samtools (version 0.1.18).

We performed these steps for 11 oesophageal samples and their matched normal samples along with an additional four normal samples obtained from other patients (Supplementary Table S1). This resulted in greater than seven million primary alignments per sample.

#### In silico generation of FAST-SeqS dilution data

We performed an in silico dilution of FAST-SeqS data by mixing sequencing reads from control samples with reads from OAC samples. Since the number of reads in the matched controls were insufficient to create samples with two million reads, we created a pool of control reads (in silico) which were used to dilute the OAC samples. This was done by subsampling two million reads from 12 control samples (which were prepared and sequenced in the same batch as the OAC samples). The total number of reads from these 12 control samples was 14,405,596. To obtain a pool of 2 million reads, we used the ‘sample’ command from seqtk (https://github.com/lh3/seqtk, version: 1.2-r101) to sample a proportion (2/14.405596) of each control sample’s reads and merged these together into a single FASTQ file. The reads that were subsampled were removed from the control samples (using a custom python script) to avoid using the same reads to fit ***m***.

We mixed the pool of control reads with the OAC samples in varying proportions to achieve a desired diluted tumour purity. The OAC samples did not have a tumour purity of 100%, instead they were themselves a mixture of tumour and normal DNA. The purity of these samples were determined by ASCAT-NGS (version 2.1) [27]. Based on ASCAT’s purity value, we calculated the number of reads required from the OAC sample to achieve a desired dilution and total number of reads. This was calculated as follows:

17 $$  \begin{aligned} &\text{required tumour reads}\\ &= \text{round} \left(\frac{\text{desired purity proportion} \cdot \text{required total reads}}{\text{ASCAT inferred purity proportion}} \right) \end{aligned}  $$

Hence, the number of control reads required were:

18 $$  {}\begin{aligned} \text{required control reads} &= \text{required total reads}\\ &\quad - \text{required tumour reads} \end{aligned}  $$

We produced in silico dilution FASTQ files in the following way:

1. We used the ‘sample’ command from seqtk to sample the required number of tumour reads from the OAC FAST-SeqS FASTQ file
2. We used the ‘sample’ command from seqtk to sample the required number of control reads from the pooled control reads FASTQ file
3. We merged the sampled tumour and control reads into a single FASTQ file

We performed these steps for each OAC sample to create diluted samples with two million total reads and the following purity values: 0.3, 0.25, 0.2, 0.15, 0.1, 0.08, 0.06, 0.05, 0.04, 0.03, 0.02, 0.01, 0.0075, 0.005, 0.0025 and 0. Here, purity is defined as the proportion of tumour reads in the sample. Of the 11 OAC samples, 8 (OAC1-7 and 9, Supplementary Table S1) were of sufficient initial tumour purity to feasibly create all the desired dilution levels.

#### In silico generation of LC WGS dilution data

We produced in silico diluted LC WGS tumour samples by mixing reads from tumour and matched normal LC WGS BAM files (previously downsampled and filtered as described above). We first calculated the number of reads in the tumour BAM and normal BAM files using samtools (samtools view -F 256 -c [BAM file]). Next, we calculated the number of reads required using Eqs. 17 and 18. Using the DownsampleSAM command (Picard tools) and the ‘HighAccuracy’ strategy, we sampled the corresponding desired proportion of reads from the tumour BAM file and normal BAM file. We used samtools to merge the resulting sampled tumour BAM file with the normal BAM file into a single file representing the diluted sample. We aimed to obtain seven million filtered primary alignments per diluted sample (as this is what we expect from nine million reads after alignment and filtering) and dilution levels which matched the diluted FAST-SeqS samples. This was performed for 8 OAC samples and their matched normals (OAC1-7 and 9).

### Processing of FAST-SeqS sequencing data to counts

Each sequencing run of the Illumina MiSeq platform produced a BCL file which was converted to FASTQ format (using Illumina’s bcl2fastq tool). Sequencing reads that failed the Illumina chastity filter were removed. The FASTQ file was demultiplexed into separate FASTQ files corresponding to each sample using the demuxFQ tool (https://github.com/gdbzork/demuxFQ) with the default settings. The sample barcodes are provided in Supplementary Table S11. Each sample’s FASTQ file was then processed through a custom pipeline which we describe below.

#### Identifying forward primer position

For each read in the FASTQ file, the position of the forward primer sequence was detected by searching for the sequence with the minimum Hamming distance to the forward primer sequence using a sliding window. Reads with a minimum Hamming distance greater than 5 were discarded.

#### Read trimming

The portion of the reads before and including the forward primer sequence were trimmed. The ends of the reads were also trimmed such that the length of the reads used for downstream analyses were 100 base pairs minus the forward primer length. Any reads shorter than 100 base pairs minus the forward primer length after trimming were discarded.

#### Quality control

After trimming, reads were discarded if they contained at least one base with a Phred quality score less than 20 and/or contained one or more ambiguous base calls (N).

#### Obtaining unique sequences and counts per unique sequence

To avoid aligning the same sequence multiple times, only unique read sequences were kept. For each unique read, the number of identical fragments were recorded.

#### Alignment of unique sequences

Unique raw read sequences were aligned with Bowtie 1.0.0 [44] (using the option: -r). Three mismatches were permitted (option: -v3) and reads aligning to multiple locations were discarded (option: -m1). The reads were aligned to GRCh38 (GenBank accession: GCA_000001405.15, no alt analysis set).

#### Counts and alignments combined

Each sample’s unique read alignments and their corresponding unique read counts were combined into a single file consisting of a matrix of counts. The rows corresponded to genomic positions (the union of genomic positions from the alignments in all samples) and columns corresponded to samples. The first three columns of the matrix corresponded to the chromosome, position and strand for the locus, respectively. The matrix of counts used in this analysis can be found in the conliga R package and in Supplementary Table S9.

### Selecting loci

Rows of the count matrix corresponding to genomic loci within chromosomes X, Y and within unplaced or unresolved contigs were discarded. For each batch of samples, genomic loci obtaining a zero count in any one of a set of control samples were also discarded. Depending on the sequencing batch we analysed and the controls chosen to filter loci (Supplementary Table S10), this resulted in approximately 10,000 - 12,000 genomic loci across chromosomes 1 to 22.

### Analysis of copy number from FAST-SeqS data using conliga

conliga (version 0.1.0) [45] was used to obtain RCN profiles for all FAST-SeqS samples in this study (Supplementary Table S1) using R (version 3.2.3) [46] and RcppAramdillo (version 0.6.500.4.0) [47]. Of the 15 OAC samples sequenced, four were excluded due to having fewer than 350,000 reads. Two control samples were excluded due to their inferred RCN profiles having two main hidden states incompatible with their supposed ‘normal’ status. The values for the priors used and MCMC settings are stated in the inference sections above. The samples used as a basis to filter loci and fit $\hat {\boldsymbol {m}}$ for each experiment are listed in Supplementary Table S10.

### Analysis of copy number from FAST-SeqS data using CNVkit

We used python 3.5.2 and CNVkit [20] version 0.9.9 to obtain RCN profiles for the OAC samples listed in Supplementary Table S1 and their corresponding in silico diluted samples using CNVkit’s batch command. The same four OAC samples and two normal samples were excluded as above. We produced a bedfile, containing the same FAST-SeqS loci used by conliga for each experiment (high-purity OAC samples and OAC in silico dilution series), i.e. those loci that had at least one read across all normal/control samples for each experiment. This bedfile was passed to CNVkit using the targets argument. The method argument was set as amplicon. For each corresponding experiment, we used the same set of control samples (listed in Supplementary Table S10) that were used by conliga as the normal reference samples. This process produced a segmented log2 ratio (.cns) file for each sample.

### Analysis of copy number from high-coverage WGS data

High-coverage WGS samples were processed and aligned using BWA-MEM [48] (version 0.5.9) and total copy number (TCN) profiles and normal contamination estimates were provided by ASCAT-NGS (version 2.1) using a pipeline previously described by Secrier et al. [9]. The only exception to this was that the reads were aligned to GRCh38 (GenBank accession: GCA_000001405.15, no alt analysis set) rather than GRCh37.

### Analysis of copy number from low-coverage WGS data

QDNAseq (version 1.6.1) was used to obtain relative copy number calls for all LC WGS data. The bin size used was 15Kb as per the analysis performed in Scheinin et al. [22] for 0.1X LC WGS. The bins were created using GRCh38 (BSgenome.Hsapiens.NCBI.GRCh38) and a mappability file (bigWig format) for 50-mers was created for GRCh38 using the GEM library (GEM-binaries-Linux-x86_64-core_i3-20130406-045632) https://sourceforge.net/projects/gemlibrary/. 15 normal LC WGS samples (Supplementary Table S1), were used to run the applyFilters and iterateResiduals functions. 11 of these 15 samples correspond to the matched normals of the oesophageal samples (Supplementary Table S1). We did not run the functions normalizeBins and normalizeSegmentedBins which scale the read counts by the median value. This was not necessary and would make the comparison between ASCAT, QDNAseq and conliga results more difficult to interpret.

### Comparison of copy number between methods

ASCAT outputs TCN in contiguous genomic regions, QDNAseq outputs *log*_2_ RCN in 15 Kb bins across the genome, CNVkit outputs *log*_2_ RCN in contiguous genomic regions, and conliga outputs RCN values at specific FAST-SeqS loci. To make a fair comparison between the tools, it was necessary to convert ASCAT’s TCN calls to RCN as follows:

19 $$  \text{RCN}_{i} = \frac{(1 - \text{normal}) \cdot \text{TCN}_{i} + \text{normal} \cdot 2}{\text{mean TCN}}  $$

Here, normal represents the estimated normal contamination value provided by ASCAT and *i* represents a contiguous genomic region or a discrete locus or fragment. In the case of a contiguous region, the mean TCN (or ploidy) was calculated as follows:

20 $$  \text{mean TCN} = \frac{ \sum_{i} \left(\text{TCN}_{i} \cdot \text{length}_{i} \right) }{\sum_{i} \text{length}_{i}}  $$

and in the case of discrete loci or fragments:

21 $$  \text{mean TCN} = \frac{ \sum_{i} \text{TCN}_{i} }{L}  $$

where *L* represents the total number of loci or fragments considered. Furthermore, we converted QDNAseq and CNVkit’s calls from *log*_2_ RCN to RCN for downstream comparison to ASCAT and conliga.

In Fig. 1f and g, we compared the RCN values at the intersection of genomic loci across ASCAT, QDNAseq, CNVkit and conliga. Since this intersection represented a subset of each method’s genomic loci, the RCN values were rescaled considering only this subset. QDNAseq, CNVkit and conliga RCN values were rescaled by the sample’s mean RCN of the considered loci. ASCAT’s RCN was calculated using Eqs. 19 and 21.

In Fig. 1h, we compared RCN values in genes of interest. Recurrently amplified and deleted genes were obtained from Dulak et al. [28] and Ross-innes et al. [15]. Here, ASCAT’s RCN values were calculated using Eqs. 19 and 20 using all called regions for each sample. For each gene in each sample, the weighted mean of the relative copy number (weighted by the length of the overlapping called region) was computed for ASCAT and QDNAseq. This was calculated as follows:

22 $$ {\text{RCN}}_{\text{gene}} = \frac{\sum_{i} {\text{RCN}}_{i} \cdot l_{i}}{\sum_{i} l_{i}}  $$

where *l*_*i*_ represents the length of the overlapping portion of the called region with the gene.

For CNVkit and conliga, we used the corresponding RCN values at FAST-SeqS loci. If loci occurred within the gene, the mean of the RCN values within the gene was used, otherwise the loci directly upstream and downstream, i.e. either side, of the gene were used, and a mean value was taken. See Supplementary Table S4 for the full list of genes used in the analysis.

#### Computing Spearman’s rank correlation

When calculating the Spearman’s rank correlation coefficient for all calls across all samples, we used the rescaled RCN value at the intersecting genomic loci between ASCAT, QDNAseq, CNVkit and conliga, using the rescaled RCN values described above for Fig. 1f and g.

## Supplementary Information

**Additional file 1** Supplementary materials (Supplementary Notes 1, 2, 3, 4 and 5. Supplementary **Table S1.** Supplementary **Fig. S1.** Supplementary **Fig. S2**).

**Additional file 2** Supplementary Table S2.

**Additional file 3** Supplementary Table S3.

**Additional file 4** Supplementary Table S4

**Additional file 5** Supplementary Table S5.

**Additional file 6** Supplementary Table S6.

**Additional file 7** Supplementary Table S7.

**Additional file 8** Supplementary Table S8.

**Additional file 9** Supplementary Table S9.

**Additional file 10** Supplementary Table S10.

**Additional file 11** Supplementary Table S11.

## Data Availability

WGS and FAST-SeqS data, and information on how to request access to these data, can be found at the European Genome-phenome Archive (EGA) under accession EGAD00001004289. The copy number results obtained from ASCAT, QDNAseq, CNVkit and conliga can be found https://osf.io/bhx6f/?view_only=ed25e2fb521d46239e5274c032350f0b. conliga source code [45] is freely available under an open-source GPLv2 license at https://github.com/samabs/conliga.

## Abbreviations

BO: Barrett’s oesophagus
CGH: Comparative genomic hybridization
ctDNA: Circulating tumour DNA
GAC: Gastric adenocarcinoma
HC WGS: High-coverage whole-genome sequencing
HDP: Hierarchical Dirichlet process
HMM: Hidden Markov model
ICGC: International Cancer Genome Consortium
LC WGS: Low-coverage whole-genome sequencing
LINE1: Long interspersed nuclear element-1
MCMC: Markov chain Monte Carlo
OAC: Oesophageal adenocarcinoma
PCR: Polymerase chain reaction
RCN: Relative copy number
SCNA: Somatic copy number alteration
SNP: Single-nucleotide polymorphism
SNV: Single-nucleotide variant
TCGA: The Cancer Genome Atlas
TCN: Total copy number
WGS: Whole-genome sequencing
