# Supplementary material for: Low-cost and clinically applicable copy number profiling using repeat DNA
Source: BMC Genomics. 2022 Aug 17;23:599. doi: 10.1186/s12864-022-08681-8 (PMC9386984; doi:10.1186/s12864-022-08681-8)
Supplement: Supplementary file 1 — Additional file 1 Supplementary materials (Supplementary Notes 1, 2, 3, 4 and 5. Supplementary Table S1. Supplementary Fig. S1. Supplementary Fig. S2). [file 12864_2022_8681_MOESM1_ESM.pdf]

Supplementary Materials: Low-cost and clinically applicable copy number profiling using repeat DNA

Abujudeh S[1,†,*], Zeki SS[2,3,†,*], van Lanschot MCJ[2], Pusung M[2], Weaver JMJ[2,4], Li X[2], Noorani A[2], Metz AJ[2], Bornschein J[2], Bower L[1], Miremadi A[2], Fitzgerald RC[2,‡,*], Morrissey ER[1,5,‡,*], and Lynch AG[1,6,‡,*]

[1]Cancer Research UK Cambridge Institute, University of Cambridge, Li Ka Shing Centre, Robinson Way, Cambridge CB2 0RE, UK

[2]Medical Research Council (MRC) Cancer Unit, University of Cambridge, Cambridge, UK

[3]Department of Gastroenterology, Guy's and St Thomas' NHS Trust, London SE1 7EH, UK

[4]Department of Medical Oncology, The Christie NHS Foundation Trust, Manchester, M20 4TX

[5]Weatherall Institute of Molecular Medicine, University of Oxford, Oxford, UK

[6]School of Mathematics and Statistics/School of Medicine, University of St Andrews, St Andrews, UK

[†]These authors contributed equally to this work

[‡]These authors contributed equally to this work

[*]Corresponding authors: samer.abujudeh@gmail.com, sebastianzeki0@gmail.com, rcf29@mrc-cu.cam.ac.uk, edward.morrissey@imm.ox.ac.uk, andy.lynch@st-andrews.ac.uk

May 25, 2022

Supplementary Note 1: Time and cost comparison for FAST-SeqS and LC WGS

Here, we provide our calculations of the cost to produce a FAST-SeqS sample and an LC WGS sample. Note that we used the prices that were available to us, and where possible this was the recommended retail price (RRP). We note that prices can vary by date, country and institution. As such, the calculations are provided as a guide and basis for relative comparison between the two sequencing approaches.

Cost of FAST-SeqS (2 million single-end 150 bp reads per sample)

The library preparation requires two rounds of PCR (see Methods) and requires deoxynucleotides (dNTPs), polymerase, RNAse free water and primers.

dNTPs

1 µL 10 mM of dNTPs are required per PCR reaction. 5 mL 10 mM (ThermoFisher Scientific) can be purchased at £317.55 at the time of writing and provides dNTPs for 5000 PCR reactions. Two PCR reactions are required per sample, equating to £0.13 per sample.

Polymerase

0.5 µL Phusion Hot Start II DNA Polymerase 2U/µL with 10 µL 5x Phusion HF Buffer is required per PCR reaction. 500 units can be purchased at £436.71 (ThermoFisher Scientific). One unit is required per PCR reaction and two PCR reactions are required per sample, equating to £1.75 per sample.

Nuclease-free water

Nuclease-free water (not DEPC-Treated) 10 x 50 mL (ThermoFisher Scientific) can be purchased at £88.28 at the time of writing. 18.5 µL - 23.5 µL is used in the first PCR reaction, depending on the amount of DNA used. This would equate to less than £0.01 per sample.

Primers

5 µL (0.5 µM) of each forward and reverse primers are required in each PCR reaction. 5 nmol of primer can be synthesized for approximately £20. Diluting with 10 mL of water gives 10 mL at 0.5 µM of primer. 5 µL of forward primer and 5 µL of reverse primer is required in each PCR reaction. Two PCR reactions are required per sample, equating to approximately £0.04 per sample.

DNA Quantification and Quality Control

Prior to pooling the samples, quantification of DNA is performed using Bioanalyzer 1000 DNA kit on an Agilent 2100 Bioanalyzer instrument, for example. 300 Bioanalyzer 1000 DNA chips can be purchased at approximately

£500, which equates to £1.67 per sample.

Next-generation sequencing

We calculated the per sample cost based on using an Illumina HiSeq 4000 sequencer which, for our purposes, produces 350 million single end (SE) 150 base pair (bp) reads from a single lane of sequencing. We factored in that our library would include 20% PhiX to increase diversity for sequencing, and as such, we would expect 280 million reads per lane to originate from our FAST-SeqS amplicons. Aiming for approximately 2 million reads per sample, this would mean multiplexing 140 FAST-SeqS on a single lane.

There is considerable variation in the cost of sequencing services, depending on sector, location, and relationship with the customer. Moreover, of those services that display their costs up front, extremely few provide a direct comparison of prices for Single-End 50bp reads and Single-End 150bp reads on an Illumina HiSeq 4000 machine. Thus, we will make use here of the Stanford Medicine Genome Servicing Sequence Centre prices, roughly converted to Sterling, obtained from http://med.stanford.edu/gssc/rates.html on 17/08/18. These costs do not differ substantially from our own experience. Thus, the cost of a single lane 150 bp SE sequencing on the HiSeq 4000 we take to be approximately £1400 at the time of writing. As such, the sequencing cost equates to £10 per sample.

Total cost per sample

The total cost for processing the samples, as explained above, is £0.13 (dNTPs) + £1.75 (polymerase) + £0.01 (nuclease-free water) + £0.04 (primers) + £1.67 (quantification and quality control) + £10 (sequencing) = £13.60.

Cost of LC WGS (9 million single-end 50 bp reads per sample)

DNA Shearing

Prior to library preparation, the input DNA needs to be sheared to a desired length distribution. This is often achieved by the use of sonification, for example using Covaris microTUBE strips. At the time of writing, 12 x 8 microTUBE strips (i.e. for 96 samples) can be purchased for £413.10 and is therefore approximately £4.30 per sample.

Library preparation

To process the DNA and prepare the library for sequencing, library preparation is required. This generally consists of end-repair, adapter ligation, and is sometimes followed by PCR amplification to generate sufficient quantities of the library for sequencing. Library preparation kits can be purchased from a variety of manufacturers, with varying costs and time to prepare each sample. Examples of library preparations include:

- Illumina TruSeq DNA PCR-Free High Throughput Library Prep Kit (96 samples) can be purchased for approximately £2,100 at the time of writing and is therefore approximately £21.88 per sample.
- KAPA Hyper Prep Kit, PCR-free (96 samples) can be purchased for approximately £1,900; £19.79 per sample
- NEBNext Ultra II DNA Library Prep Kit for Illumina (E7103L, 96 reactions): £1,986; £20.69 per sample.
- SMARTer ThruPLEX DNA-seq 96D Kit (R400407, 96 reactions); £4,063; £42.32 per sample

DNA Quantification and Quality Control

Similarly to FAST-SeqS, quantification of DNA is performed using Bioanalyzer 1000 DNA kit on an Agilent 2100 Bioanalyzer instrument, for example. 300 Bioanalyzer 1000 DNA chips can be purchased at approximately £500, which equates to £1.67 per sample.

Next-generation sequencing

We calculated the per sample cost based on using an Illumina HiSeq 4000 sequencer, which can be used for low-coverage WGS to produce 350 million single end (SE) 50 base pair (bp) reads from a single lane of sequencing. PhiX should not be required as the library should not be low complexity. To achieve approximately 9 million reads per sample in order to obtain approximately 0.1X coverage as per Scheinin et al. [1], would mean multiplexing 38 samples on a single lane. The cost of a single lane 50 bp SE sequencing on the HiSeq 4000 we take to be £1000 (justification as in the previous section). As such, the sequencing cost equates to ∼£26 per sample.

Total cost per sample

The total cost for processing a low-coverage WGS sample (0.1X coverage, single-end 50 bp reads), as explained above is, £4.30 (DNA shearing) + approximately £20-£40 (library preparation) + £1.67 (quantification and quality control) + £26 (sequencing) = £52-£72.

Supplementary Note 2: Aspects of FAST-SeqS data

We explored the loci counts of normal samples (which were assumed to be predominantly diploid) and observed various aspects of the data which led to the model. We observed (1) a technical bias in the number of reads aligned to each locus (Supplementary Figure 1b and 1c), (2) the variation in the data exceeds that expected from sampling variation alone (over-dispersion) (Supplementary Figure 1d), (3) this additional variation is likely to be predominantly technical and the amount of this variation varies between samples, (4) the expected proportion of reads at each locus is directly proportional to the relative copy number between loci (Supplementary Figure

1e), and (5) the genomic distance between loci implies that neighboring loci are likely to share the same copy number (Supplementary Figure 1f).

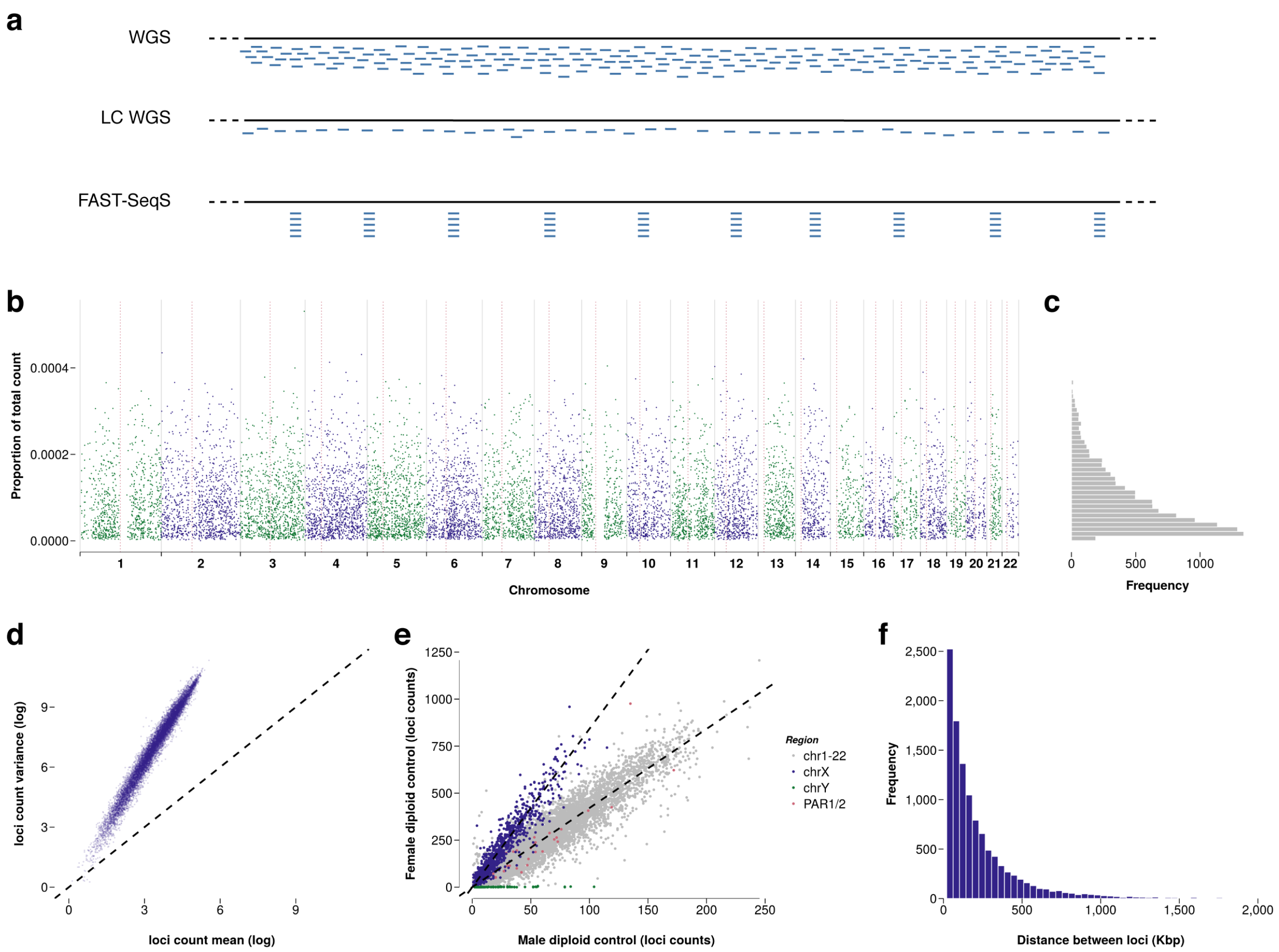

Supplementary Figure 1: Aspects of FAST-SeqS data. (a) a graphical representation of the different approaches to sequencing for the purposes of SCNA profiling; high-coverage WGS (top), low-coverage WGS (middle), FAST-SeqS (bottom). (b) The proportion of reads obtained at each locus in chr1-22 for control sample (NORM1). (c) Histogram of the proportion of reads obtained at each locus across in chr1-22 for control sample NORM1. (d) log mean vs log variance for each locus in control samples. (e) A male control sample (NORM2) counts plotted against a female control sample (NORM1) counts, showing a relative doubling of count proportions in chrX for the female control sample vs male and absence of counts from chrY in the female sample. (f) Histogram of distances between loci, with a mean distance of approximately 200Kbp between loci.

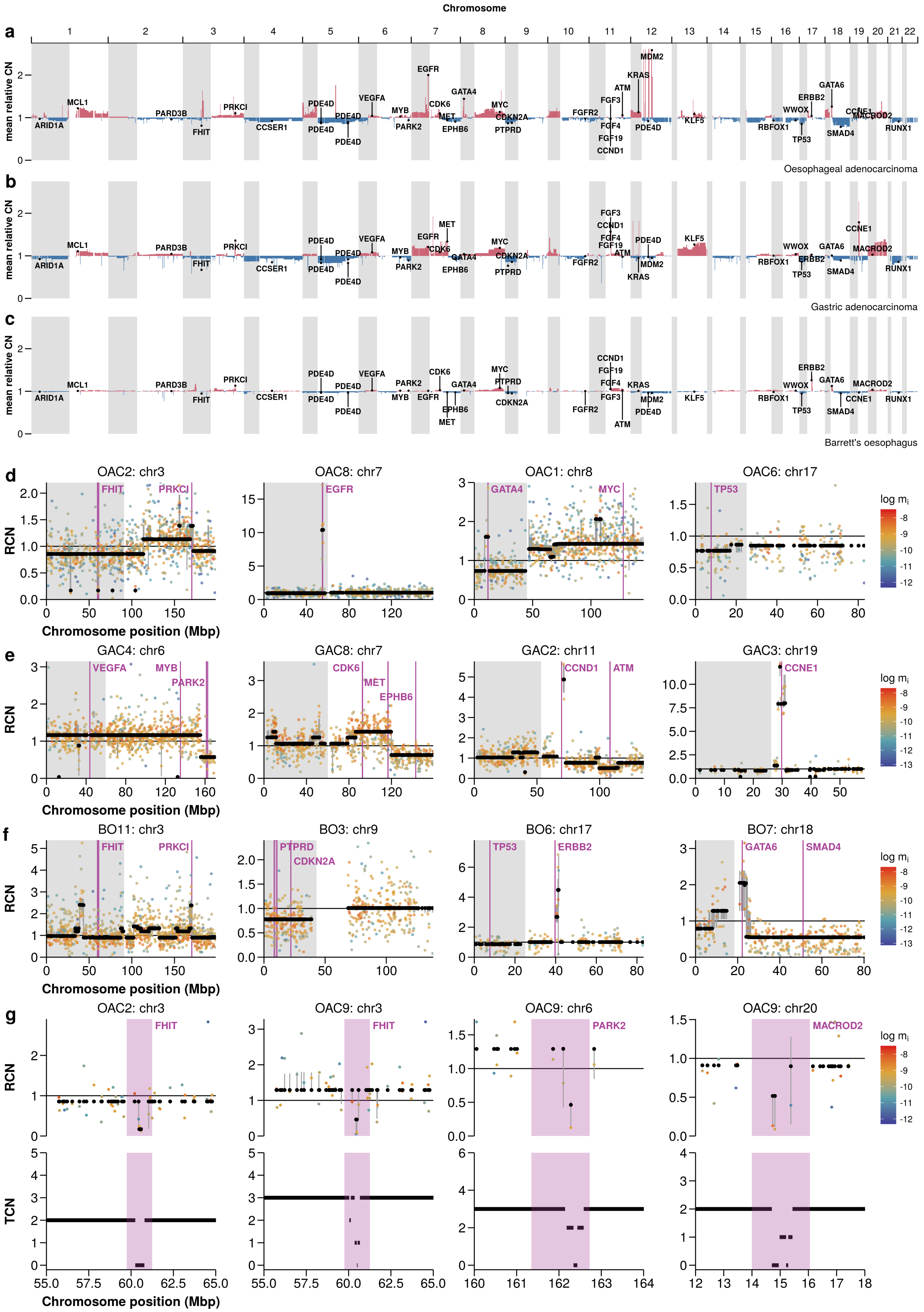
Chromosome
a
b
c
d
e
f
g
mean relative CN
Oesophageal adenocarcinoma
Gastric adenocarcinoma
Barrett's oesophagus
ARID1A
MCL1
PARD3B
FHIT
PRKCI
CCSER1
PDE4D
VEGFA
MYB
PARK2
EGFR
CDK6
MET
EPHB6
GATA4
MYC
CDKN2A
PTPRD
FGFR2
FGF3
FGF4
FGF19
CCND1
ATM
KRAS
MDM2
KLF5
RBFOX1
WWOX
TP53
ERBB2
GATA6
SMAD4
CCNE1
MACROD2
RUNX1
OAC2: chr3
OAC8: chr7
OAC1: chr8
OAC6: chr17
GAC4: chr6
GAC8: chr7
GAC2: chr11
GAC3: chr19
BO11: chr3
BO3: chr9
BO6: chr17
BO7: chr18
OAC9: chr3
OAC9: chr6
OAC9: chr20
RCN
TCN
log m_i
Chromosome position (Mbp)

Supplementary Figure 2: Copy number profile summary of patient cohorts used in this study. (a) Mean relative copy number profile for 11 oesophageal adenocarcinoma samples. (b) Mean relative copy number profile for 8 gastric adenocarcinoma samples. (c) Mean relative copy number profile for 16 Barrett's oesophagus samples, with varying levels of dysplasia. (d)-(f) Examples of relative copy number profiles for various chromosomes from different samples for OAC, GAC and BO respectively. Black points represent the maximum a posteriori (MAP) relative copy number for each locus, the coloured points represent the proportion of reads expected in a control sample (log), with red representing a high proportion and blue representing a low proportion, grey lines represent 90% credible intervals. (g) Zoomed-in regions of chromosomes 3, 6 and 20 showing intra-gene deletion of FHIT, PARK2 and MACROD2. conliga results (top) with comparison to ASCAT (bottom).

Supplementary Table 1: Variables used in the model and MCMC algorithms

Variable	Description
$z_{r,l}$	Index of hidden state that generated observation $y_{r,l}$ in locus l of chromosome arm r
$y_{r,l}$	Count observation at locus l in chromosome arm r for sample with copy number profile $\boldsymbol{z}$
$x_{k,r,l}$	Count observation at locus l in chromosome arm r for control sample k
$m_{r,l}$	Probability of an aligned sequencing read originating from locus l in chromosome arm r in a control sample
$\hat{m}_{r,l}$	Maximum a posteriori (MAP) estimate of $m_{r,l}$
$\tilde{\theta}_{r,l}$	Probability of observing an aligned read at locus l in chromosome arm r for sample with copy number profile $\boldsymbol{z}$
$\theta_{r,l}$	Probability of observing an aligned read at locus l in chromosome arm r for control sample, k
$\tilde{n}$	Total number of counts for sample with unknown copy number profile. $\tilde{n} = \sum_{r\in\mathcal{R}} \sum_{l}^{L_r} y_{r,l}$
n_k	Total number of counts for control sample k. $n = \sum_{r\in\mathcal{R}} \sum_{l}^{L_r} x_{r,l,k}$
$\tilde{s}$	Precision parameter for sample with unknown copy number profile
s_k	Precision parameter for control sample k
$\pi_{u,v}$	Transition probability from hidden state u to hidden state v
π_u^0	Initial probability of being in state u, i.e. probability of $z_{*,1} = u$
$\hat{c}_u$	Relative copy number for hidden state u
H	Base distribution for the copy number states (Gamma distribution with parameters $\boldsymbol{\lambda}$)
$\boldsymbol{\lambda}$	Parameters of the Gamma base distribution for the relative copy number states
$\boldsymbol{\omega}$	Parameters for the Gamma distribution prior on the inverse dispersion parameter for sample with copy number profile $\boldsymbol{z}$
$\boldsymbol{\psi}$	Parameters for the Gamma distribution prior on the inverse dispersion parameter for control samples
$\boldsymbol{\phi}$	Parameters for the Beta distribution prior on the expected proportions, $\boldsymbol{m}$

α	Hyperparameter of the sticky HDP-HMM
κ	Hyperparameter of the sticky HDP-HMM: the self-transition parameter
γ	Hyperparameter of the sticky HDP-HMM: the greater the value of γ the greater expected number of hidden states
β	The global transition distribution vector
ρ	Defined as $\frac{\kappa}{\alpha+\kappa}$
$\boldsymbol{A}$	Vector representing hyperparameter priors for $(\alpha+\kappa)$, γ and ρ. $A_{(\alpha+\kappa),a}$ and $A_{(\alpha+\kappa),b}$ are the shape and rate parameters of the Gamma prior distribution on $(\alpha+\kappa)$ respectively. $A_{\gamma,a}$ and $A_{\gamma,b}$ are the shape and rate parameters of the Gamma prior distribution on γ respectively. $A_{\rho,c}$ and $A_{\rho,d}$ are the shape parameters of the Beta prior distribution on ρ
L_r	The number of loci in chromosome arm r, where $r \in \mathcal{R}$
$\mathcal{R}$	The set of chromosome arms of interest, for example: $\mathcal{R} = \{\text{chr1p}, \text{chr1q}, \ldots \text{chr22p}, \text{chr22q}, \text{chrXp}, \text{chrXq}, \text{chrYp}, \text{chrYq}\}$
K	The number of control samples
σ_m	The standard deviation for the (Normal) proposal distribution for proposed values of $m_{r,l}$ in algorithm 1
σ_s	The standard deviation for the (Normal) proposal distribution for proposed values of s_k in algorithm 1
$\sigma_{\tilde{s}}$	The standard deviation for the (Normal) proposal distribution for proposed values of $\tilde{s}$ in algorithm 2
$\sigma_{\hat{c}}$	The standard deviation for the (Normal) proposal distribution for proposed values of $\hat{c}_u$ in algorithm 2
S	The fixed truncation level for the Dirichlet approximation to the Dirichlet Process.
$\boldsymbol{T}$	Matrix of state transitions counts, where $T_{u,v}$ represents the number of transitions from state u to v in the current iteration of the MCMC
$\boldsymbol{T}^0$	Vector of state counts for locus 1 of all chromosome arms, where T^0_u represents the number of loci (at the beginning of a chromosome arm) assigned to state u in the current iteration of the MCMC
N	The total number of iterations in the MCMC
$\boldsymbol{M}, \boldsymbol{W}, \bar{\boldsymbol{M}}$	

$\boldsymbol{h}, \boldsymbol{g}, \bar{K}, \zeta, \eta$ Auxiliary variables used to sample $(\alpha + \kappa)$, γ and ρ

Supplementary Note 3: Further details of generative model

Full generative model

Here we describe the full generative model for the loci counts, $\boldsymbol{x}$, of K control samples together with the loci counts, $\boldsymbol{y}$, of a non-control sample with copy number profile $\hat{c}_{z_{r,l}}$ for all r and l, along with all latent variables. This could be extended to include all J non-control samples, so that β, π^0, π_u, $\hat{c}_u$, $z_{r,l}$, $\tilde{s}$, $\tilde{\theta}_{r,l}$, $y_{r,l}$ and $\tilde{n}$ are sample specific and include an index j. However, here we drop the j index and consider only one non-control sample for simplicity.

$$
\begin{aligned}
\beta \mid \gamma &\sim \mathrm{GEM}(\gamma) \\
\pi^0 \mid \alpha, \beta &\sim \mathrm{DP}\left(\alpha, \beta\right) \\
\pi_u \mid \beta, \alpha, \kappa &\sim \mathrm{DP}\left(\alpha + \kappa, \frac{\alpha\beta + \kappa\delta_u}{\alpha + \kappa}\right) \\
\hat{c}_u \mid H, \lambda &\sim H(\lambda) \\
z_{r,1} \mid \pi^0 &\sim \pi^0 \\
z_{r,l} \mid \{\pi_u\}_{u=1}^{\infty}, z_{r,l-1} &\sim \pi_{z_{r,l-1}}, \text{ for } l > 1 \\
\tilde{s} \mid \omega &\sim \mathrm{Gamma}(\omega_{\text{shape}}, \omega_{\text{scale}}) \\
s_k \mid \omega &\sim \mathrm{Gamma}(\omega_{\text{shape}}, \omega_{\text{scale}}) \\
m_{r,l} \mid \phi_{c,r,l}, \phi_{d,r,l} &\sim \mathrm{Beta}(\phi_{c,r,l}, \phi_{d,r,l}) \\
\theta_{r,l,k} \mid s_k, m_{r,l} &\sim \mathrm{Beta}(s_k m_{r,l}, s_k(1 - m_{r,l})) \\
x_{r,l,k} \mid \theta_{r,l,k}, n_k &\sim \mathrm{Binomial}(n_k, \theta_{r,l,k}) \\
\tilde{\theta}_{r,l} \mid \{\hat{c}_u\}_{u=1}^{\infty}, z_{r,l}, \hat{m}_{r,l}, \tilde{s} &\sim \mathrm{Beta}(\tilde{s}\hat{c}_{z_{r,l}}\hat{m}_{r,l}, \tilde{s}(1 - \hat{c}_{z_{r,l}}\hat{m}_{r,l})) \\
y_{r,l} \mid \tilde{\theta}_{r,l}, \tilde{n}, &\sim \mathrm{Binomial}(\tilde{n}, \tilde{\theta}_{r,l})
\end{aligned} \tag{1}
$$

See Supplementary Table 1 and Methods for an explanation of the variables. Note that this model is not implemented but is shown for completeness (see below for how we split this generative model into two separate generative models in practice). Notice that we assume that the sample inverse dispersion parameters are drawn from the same distribution, reflecting our belief that normal and tumor count observations should have the same level of noise. Here we assume fixed values for $\boldsymbol{\omega}$. If we were to use this model as a basis for inference of the latent variables, we may wish to place priors over the values of $\boldsymbol{\omega}$ and infer them from the data.

Split generative model (for more efficient inference)

We decided to split the full model in two parts. The first part describes the generation of the data for the control samples. The second part describes the generation of the copy number profile and loci count observations for a non-control sample, using the maximum a prior (MAP) estimate of $\boldsymbol{m}$. We did this to allow for simpler implementation of the inference algorithms. In this way, we could infer the MAP estimates of $\boldsymbol{m}$ and the nuisance parameters $\boldsymbol{s}$ using the counts of the control samples. Once MAP estimates were obtained, we could infer the copy number profile for each non-control sample in independent MCMC chains, allowing the inference of each sample to be run in parallel.

Part 1: Generative model for control counts

$$\begin{aligned} s_k \mid \psi &\sim \text{Gamma}(\psi_{\text{shape}}, \psi_{\text{scale}}) \\ m_{r,l} \mid \phi &\sim \text{Beta}(\phi_{c,r,l}, \phi_{d,r,l}) \\ \theta_{r,l,k} \mid s_k, m_{r,l} &\sim \text{Beta}(s_k m_{r,l}, s_k(1 - m_{r,l})) \\ x_{r,l,k} \mid \theta_{r,l,k}, n_k &\sim \text{Binomial}(n_k, \theta_{r,l,k}) \end{aligned} \tag{2}$$

Part 2: Generative model for the counts of a sample with a relative copy number profile

$$\begin{aligned} \beta \mid \gamma &\sim \text{GEM}(\gamma) \\ \pi^0 \mid \alpha, \beta &\sim \text{DP}\left(\alpha, \beta\right) \\ \pi_u \mid \alpha, \kappa, \beta &\sim \text{DP}\left(\alpha + \kappa, \frac{\alpha\beta + \kappa\delta_u}{\alpha + \kappa}\right) \\ \hat{c}_u \mid H, \lambda &\sim H(\lambda) \\ z_{r,1} \mid \pi^0 &\sim \pi^0 \\ z_{r,l} \mid \{\pi_u\}_{u=1}^{\infty}, z_{r,l-1} &\sim \pi_{z_{r,l-1}}, \text{ for } l > 1 \\ \tilde{s} \mid \omega &\sim \text{Gamma}(\omega_{shape}, \omega_{scale}) \\ \tilde{\theta}_{r,l} \mid \{\hat{c}_u\}_{u=1}^{\infty}, z_{r,l}, \hat{m}_{r,l}, \tilde{s} &\sim \text{Beta}(\tilde{s}\hat{c}_{z_{r,l}}\hat{m}_{r,l}, \tilde{s}(1 - \hat{c}_{z_{r,l}}\hat{m}_{r,l})) \\ y_{r,l} \mid \tilde{\theta}_{r,l}, \tilde{n}, &\sim \text{Binomial}(\tilde{n}, \tilde{\theta}_{r,l}) \end{aligned} \tag{3}$$

Note that here we allow the distribution of sample specific parameters to vary between the normals (in part 1) and the sample with relative copy number profile $\boldsymbol{z}$ (in part 2). This distinction is made so that we can use the posterior distribution of s_k to update our prior distribution over $\tilde{s}$.

Supplementary Note 4: Notation used in MCMC algorithms

We denote the probability mass function (pmf) of the compound Beta-Binomial distribution as follows:

$$f_{\text{BB}}(k; n, a, b) = \binom{n}{k} \frac{\text{B}(k + a, n - k + b)}{\text{B}(a, b)} \tag{4}$$

where B represents the Beta function, n represents the total number of Bernoulli trials (counts), k represents a count observation ($k \in \{0, \ldots, n\}$), a and b are parameters ($a > 0$, $b > 0$). This is used as our likelihood function in algorithm 1 where $k = x_{r,l,k}$, $n = n_k$, $a = s_k m_{r,l}$, and $b = s_k(1 - m_{r,l})$. It is also used as a likelihood function in algorithm 2 where $k = y_{r,l}$, $n = \tilde{n}$, $a = \tilde{s}\hat{m}_{r,l}$, and $b = \tilde{s}(1 - \hat{m}_{r,l})$.

We denote the probability density function (pdf) of the Beta distribution as follows:

$$f_{\text{Beta}}(x; c, d) = \frac{1}{\mathrm{B}(c, d)} x^{c-1}(1 - x)^{d-1} \tag{5}$$

where x represents an observation ($x \in [0, 1]$), and c and d are the shape parameters of the Beta distribution ($c > 0$, $d > 0$).

We denote the pdf of the Gamma distribution as:

$$f_{\text{Gamma}}(x; k, \theta) = \frac{1}{\Gamma(k)\theta^k} x^{k-1} e^{-\frac{x}{\theta}} \tag{6}$$

where Γ represents the Gamma function, k is the shape parameter ($k > 0$), θ is the scale parameter ($\theta > 0$) and observation x (where $x \geq 0$).

The normal distribution is denoted $N(\mu, \sigma)$ and the uniform distribution is denoted $U(a, b)$.

We use the follow notation:

- $x_{\cdot,j} = \sum_i x_{i,j}$
- $x_{i,\cdot} = \sum_j x_{i,j}$
- $x_{i,*}$ denotes the ith row vector of matrix, x
- $x_{*,j}$ denotes the jth column vector of matrix, x
- $x_{i,\setminus j} = \{x_{i,1}, \ldots, x_{i,j-1}, x_{i,j+1}, \ldots x_{i,J}\}$, where J is largest value of j
- $|X|$ represents the cardinality of set X

Supplementary Note 5: MCMC for sampling posterior distributions of $\boldsymbol{m}$ and $\boldsymbol{s}$ using counts from control samples

Algorithm 1 Inferring the expected proportions of reads, $\mathbf{m}$, for each locus and the sample precision values, s_k for diploid controls $1, \ldots, K$

```
procedure INFERBBPARAMS(x, φ, ψ, N, σ_m, σ_s)                                  ▷
  Initialise MCMC:
  for each chromosome arm, r, do
    for each locus, l, in chromosome arm, r, do
      Initialise m_{r,l} to x_{·,r,l} / x_{·,·,·}
  for each control sample, k, do
    Draw sample precision, s_k, from Gamma(ψ_shape, ψ_scale)
  Begin MCMC:
  for each iteration, 1 to N do
    Update expected proportions, m:
    for each chromosome arm, r, do
      for each locus, l, in chromosome arm, r, do
        Propose new value for expected proportion: m*_{r,l} ~ N(m_{r,l}, σ_m)
        if m*_{r,l} > 0 then
          p_new ← Σ_k (log f_BB(x_{k,r,l}; n_k, s_k m*_{r,l}, s_k(1 − m*_{r,l}))) + log f_Beta(m*_{r,l}; φ_c, φ_d)
          p_old ← Σ_k (log f_BB(x_{k,r,l}; n_k, s_k m_{r,l}, s_k(1 − m_{r,l}))) + log f_Beta(m_{r,l}; φ_c, φ_d)
          p ~ U(0, 1)
          if exp(p_new − p_old) < p then
            Accept new value: m_{r,l} = m*_{r,l}
    Update sample precision values, s:
    for each control sample, k, do
      Propose new value for sample precision: s*_k ~ N(s_k, σ_s)
      p_new ← Σ_r Σ_l (log f_BB(x_{k,r,l}; n_k, s*_k m_{r,l}, s*_k(1 − m_{r,l}))) + log f_Gamma(s*_k; ψ_shape, ψ_scale)
      p_old ← Σ_r Σ_l (log f_BB(x_{k,r,l}; n_k, s_k m_{r,l}, s_k(1 − m_{r,l}))) + log f_Gamma(s_k; ψ_shape, ψ_scale)
      p ~ U(0, 1)
      if exp(p_new − p_old) < p then
        Accept new value: s_k = s*_k
```

Supplementary Note 6: MCMC for sampling posterior distributions of $\hat{\boldsymbol{c}}$, $\boldsymbol{z}$ and $\tilde{s}$ using the counts from a sample with unknown copy number profile

Algorithm 2 Inferring the relative copy number profile and precision, $\tilde{s}$, of a new sample

```
procedure InferRCN(y, A, ω, λ, m̂, L, ñ, N, S, σ_ĉ, σ_s̃)
    Initialise MCMC:
    If γ not fixed, draw γ from prior: γ ~ Gamma(A_{γ,a}, A_{γ,b})
    If (α + κ) not fixed, draw (α + κ) from prior: α + κ ~ Gamma(A_{(α+κ),a}, A_{(α+κ),b})
    If ρ not fixed, draw ρ from prior: ρ ~ Beta(A_{ρ,c}, A_{ρ,d})
    Set κ ← ρ(α + κ)
    Set α ← (α + κ) − κ
    Draw sample precision, s̃, from prior: s̃ ~ Gamma(ω_shape, ω_scale)
    for each chromosome arm, r, do
        for each locus, l, in chromosome arm, r, do
            If not initialized, then randomly draw hidden state: z_{r,l} ~ Categorical(S, {1/S, ..., 1/S})
    T^0, T ← countTransitions(z, L, S)
    Define transition probabilities, π, as a S by S matrix
    Define initial distribution, π^0, as a vector of length S
    Set π and π^0 as follows:
    for each state, u, from 1 to S, do
        Set π^0_u ← T^0_u / Σ_u T^0
        if T_{·u} is equal to 0 then
            Set π_{u,u} ← 1, π_{u,\u} ← 0
        else
            for each state, v, from 1 to S, do
                Set π_{u,v} ← T_{u,v} / T_{·,v}
    for each state, u, from 1 to S do
        If not initialized, draw relative copy number for state u: c_u ~ Gamma(λ_shape, λ_scale)
    Draw β via the stick breaking (GEM) process as follows:
    initialise len to 1 and initialise i to 1
    while i ≤ S do
        β_i ~ Beta(1, γ) · len
        len ← len − β_i
        increment i
    Begin MCMC
    for each iteration, 1 to N do
        q ← computeLogLikCache(y, m̂, ĉ, ñ, s̃, S)
        for each chromosome arm, r, do
            μ ← computeMessages(q, π, L_r, r, S)
            z_{r,*} ← sampleStates(q, μ, π, π^0, L_r, r)
        T^0, T ← countTransitions(z, L, S)
        M, w, M̄ ← sampleAuxVars(β, T, α, κ, S)
        Sample Global Transition Distribution: β ~ Dir(γ/S + M̄_{·,1}, ..., γ/S + M̄_{·,S})
        π^0, π ← sampleTransDists(T^0, T, S)
        ĉ ← sampleCopyNumber(z, ĉ, m̂, y, λ, q, S, s̃, ñ, σ_ĉ)
        s̃ ← samplePrecision(y, ĉ, m̂, L, ω, R, ñ, σ_s̃)
        α, κ, γ ← sampleHyperparameters(T, M, W, M̄, A, α, κ, γ, S)
```

function COMPUTELOGLIKCACHE($\boldsymbol{y}, \hat{\boldsymbol{m}}, \hat{\boldsymbol{c}}, \tilde{n}, \tilde{s}, S$)

▷ This function is used so that we do not needlessly calculate the log likelihood function several times during an iteration of the MCMC.

for `each chromosome arm,` r**, do**
  for `each locus,` l`, in chromosome arm,` r**, do**
    for `each state,` u`, from 1 to` S**, do**
      Store log likelihood for locus in state:
      $q_{r,l,u} \leftarrow \log f_{BB}(y_{r,l}; \tilde{n}, \tilde{s}\hat{c}_u\hat{m}_{r,l}, \tilde{s}(1 - \hat{c}_u\hat{m}_{r,l}))$
return $\boldsymbol{q}$

function COMPUTEMESSAGES($\boldsymbol{q}, \boldsymbol{\pi}, L_r, r, S$) ▷ Note that $\mu_{l,u}$ denotes the backward message passed from z_{l+1} to z_l

for `each state,` $u \in \{1, \ldots, S\}$ **do**
  Initialise messages:
  $\mu_{L_r,u} \leftarrow 1$
for `each locus,` $l \in \{L_r - 1, \ldots, 1\}$ **do**
  for `each state,` $u \in \{1, \ldots, S\}$ **do**
    $\mu_{l,u} \leftarrow \sum_{v=1}^{S} (\pi_{u,v} \cdot \exp(q_{r,l,v}) \cdot \mu_{l+1,v})$
  Scale row of messages by the maximum value:
  $\boldsymbol{\mu}_{l,*} \leftarrow \boldsymbol{\mu}_{l,*} / \max_{1 \le u \le S} \mu_{l,u}$
return $\boldsymbol{\mu}$

function SAMPLESTATES($\boldsymbol{q}, \boldsymbol{\mu}, \boldsymbol{\pi}, \boldsymbol{\pi}^0, L_r, r$)

for `each state,` $u \in \{1, \ldots, S\}$ **do**
  $\log p_u \leftarrow \log \pi_u^0 + \log \mu_{1,u} + q_{r,1,u}$
Scale $\boldsymbol{p}$ to avoid underflow: $\boldsymbol{p} \leftarrow \exp(\log \boldsymbol{p} - \max_{1 \le u \le S} \log p_u)$
Make $\boldsymbol{p}$ sum to 1: $\boldsymbol{p} \leftarrow \boldsymbol{p} / \sum_{u=1}^{S} p_u$
Sample state at the first locus of chromosome arm: $z_1 \sim \text{Categorical}(S, \boldsymbol{p})$
for `each locus,` $l \in \{2, \ldots, L_r\}$ **do**
  for `each state,` $u \in \{1, \ldots, S\}$ **do**
    $\log p_u \leftarrow \log \pi_{z_{l-1},u} + \log \mu_{l,u} + q_{r,l,u}$
  Scale $\boldsymbol{p}$ to avoid underflow: $\boldsymbol{p} \leftarrow \exp(\log \boldsymbol{p} - \max_{1 \le u \le S} \log p_u)$
  Make $\boldsymbol{p}$ sum to 1: $\boldsymbol{p} \leftarrow \boldsymbol{p} / \sum_{u=1}^{S} p_u$
  Sample state at locus, l, of chromosome arm: $z_l \sim \text{Categorical}(S, \boldsymbol{p})$
return $\boldsymbol{z}$

function SAMPLEAUXVARS($\boldsymbol{\beta}, \boldsymbol{T}, \alpha, \kappa, S$)

Sample $\boldsymbol{M}$:
for `each state,` $u \in \{1, \ldots, S\}$ **do**
  for `each state,` $v \in \{1, \ldots, S\}$ **do**
    Set $M_{u,v} \leftarrow 0$
    for $i \in \{1, \ldots, T_{u,v}\}$ **do**
      sample: $a \sim \text{Bernoulli}\left(\frac{\alpha\beta_v + \kappa\delta(u,v)}{i + \alpha\beta_v + \kappa\delta(u,v)}\right)$
      if a is equal to 1 **then**
        increment $M_{u,v}$
Sample $\boldsymbol{W}$:
Set $\rho \leftarrow \frac{\kappa}{\alpha + \kappa}$
for `each state,` $u \in \{1, \ldots, S\}$ **do**
  $W_{u,\cdot} \sim \text{Binomial}\left(M_{u,u}, \frac{\rho}{\rho + \beta_u(1-\rho)}\right)$
Set $\bar{\boldsymbol{M}}$:

$$\bar{M}_{u,v} = \begin{cases} M_{u,v}, & u \neq v; \\ M_{u,u} - W_{u,\cdot}, & u = v \end{cases}$$

return $\boldsymbol{M}, \boldsymbol{W}, \bar{\boldsymbol{M}}$ ▷ here $\boldsymbol{w}$ represents the vector $(W_{1,\cdot}, \ldots, W_{S,\cdot})$

function COUNTTRANSITIONS($\boldsymbol{z}, \boldsymbol{L}, S$)
    Set transition counts, $\boldsymbol{T}$, to a S by S matrix of zeros
    Set initial state counts, $\boldsymbol{T}^0$, to a vector of zeros of length S
    Update $\boldsymbol{T}$ and $\boldsymbol{T}^0$ as follows:
    for `each chromosome arm,` r**,** **do**
        increment $T^0(z_{r,1})$
        for `locus,` l`, to` $L_r - 1$`, in chromosome arm,` r**,** **do**
            increment $T(z_{r,l}, z_{r,l+1})$
    return $\boldsymbol{T}^0, \boldsymbol{T}$

function SAMPLETRANSDISTS($\boldsymbol{T}^0, \boldsymbol{T}, S$)   ▷ Sample $\boldsymbol{\pi}^0$ and $\boldsymbol{\pi}$
    Sample the initial distribution:
    $\boldsymbol{\pi}^0 \sim \text{Dirichlet}\left(\alpha\beta_1 + T_1^0, \ldots, \alpha\beta_S + T_S^0\right)$
    Sample the transition matrix:
    for `each state,` $u \in \{1, \ldots, S\}$ **do**
        Sample row u of the transition matrix:
        $\boldsymbol{\pi}_{u,*} \sim \text{Dirichlet}\left(\alpha\beta_1 + T_{u,1}, \ldots, \alpha\beta_u + \kappa + T_{u,u}, \ldots, \alpha\beta_S + T_{u,S}\right)$
    return $\boldsymbol{\pi}^0, \boldsymbol{\pi}$

function SAMPLECOPYNUMBER($\boldsymbol{z}, \hat{\boldsymbol{c}}, \hat{\boldsymbol{m}}, \boldsymbol{y}, \boldsymbol{\lambda}, \boldsymbol{q}, S, \tilde{s}, \tilde{n}, \sigma_{\hat{c}}$)
    Sample the copy number of each state in turn:
    for `each state,` $u \in \{1, \ldots, S\}$ **do**
        Select the loci counts and expected proportions in state u:
        $Y_u = \{y_{r,l} \mid z_{r,l} = u\}$
        $\hat{M}_u = \{\hat{m}_{r,l} \mid z_{r,l} = u\}$
        if $|Y_u| \neq 0$ **then**   ▷ If there are loci assigned to state u
            Propose new value for relative copy number for state u:
            $\hat{c}_u^* \sim N(\hat{c}_u, \sigma_{\hat{c}})$
            if $\hat{c}_u > 0$ **then**
                $p_{\text{old}} \leftarrow \sum_{i=1}^{|Y_u|} \left(\log f_{BB}(Y_i; \tilde{n}, \tilde{s}\hat{c}_u\hat{M}_i, \tilde{s}(1 - \tilde{s}\hat{c}_u\hat{M}_i)\right) + \log f_{\text{Gamma}}(\hat{c}_u; \lambda_{\text{shape}}, \lambda_{\text{scale}})$
                $p_{\text{new}} \leftarrow \sum_{i=1}^{|Y_u|} \left(\log f_{BB}(Y_i; \tilde{n}, \tilde{s}\hat{c}_u^*\hat{M}_i, \tilde{s}(1 - \tilde{s}\hat{c}_u^*\hat{M}_i)\right) + \log f_{\text{Gamma}}(\hat{c}_u^*; \lambda_{\text{shape}}, \lambda_{\text{scale}})$
                $p \sim U(0, 1)$
                if $\exp(p_{\text{new}} - p_{\text{old}}) < p$ **then**
                    Accept new value: $\hat{c}_u = \hat{c}_u^*$
        else   ▷ No loci assigned to state u
            Draw new value from Base Distribution:
            $\hat{c}_u \sim \text{Gamma}(\lambda_{\text{shape}}, \lambda_{\text{scale}})$
    return $\hat{\boldsymbol{c}}$

function SAMPLEPRECISION($\boldsymbol{y}, \hat{\boldsymbol{c}}, \hat{\boldsymbol{m}}, \boldsymbol{L}, \boldsymbol{\omega}, \mathcal{R}, \tilde{n}, \sigma_{\tilde{s}}$)
    Propose new value of precision:
    $\tilde{s}^* \leftarrow N(\tilde{s}, \sigma_{\tilde{s}})$
    if $\tilde{s}^* > 0$ **then**
        $p_{\text{old}} \leftarrow \sum_{r \in \mathcal{R}} \sum_{l=1}^{L_r} \left(\log f_{BB}(y_{r,l}; \tilde{n}, \tilde{s}\hat{c}_{z_{r,l}}\hat{m}_{r,l}, \tilde{s}(1 - \hat{c}_{z_{r,l}}\hat{m}_{r,l}))\right) + \log f_{\text{Gamma}}(\tilde{s}; \omega_{\text{shape}}, \omega_{\text{scale}})$
        $p_{\text{old}} \leftarrow \sum_{r \in \mathcal{R}} \sum_{l=1}^{L_r} \left(\log f_{BB}(y_{r,l}; \tilde{n}, \tilde{s}^*\hat{c}_{z_{r,l}}\hat{m}_{r,l}, \tilde{s}^*(1 - \hat{c}_{z_{r,l}}\hat{m}_{r,l}))\right) + \log f_{\text{Gamma}}(\tilde{s}^*; \omega_{\text{shape}}, \omega_{\text{scale}})$
        $p \sim U(0, 1)$
        if $exp(p_{\text{new}} - p_{\text{old}}) < p$ **then**
            Accept new value: $\tilde{s} = \tilde{s}^*$
    return $\tilde{s}$

function SAMPLEHYPERPARAMETERS($\boldsymbol{T}, \boldsymbol{M}, \boldsymbol{W}, \bar{\boldsymbol{M}}, \boldsymbol{A}, \alpha, \kappa, \gamma, S$) ▷ Optionally sample hyperparameters (if fixed values not provided)

If not provided as a fixed value, sample $(\alpha + \kappa)$:

First, sample auxiliary variables, $\boldsymbol{h}$ and $\boldsymbol{g}$:

for `each state,` $u \in \{1, \ldots, S\}$ **do**

$h_u \sim \text{Bernoulli}\left(\frac{T_{u,\cdot}}{T_{u,\cdot}+\alpha+\kappa}\right)$

$g_u \sim \text{Beta}\left(\alpha + \kappa + 1, T_{u,\cdot}\right)$

Then sample $(\alpha + \kappa)$ as follows:

$(\alpha + \kappa) \sim \text{Gamma}\left(A_{(\alpha+\kappa),a} + M_{\cdot,\cdot} - \sum_{u=1}^{S} h_u, A_{(\alpha+\kappa),b} - \sum_{u=1}^{S} \log g_u\right)$

If not provided as a fixed value, sample γ:

Set $\bar{K} \leftarrow \sum_{u=1}^{S} \mathbb{1}(\bar{M}_{\cdot,u} > 0)$, where $\mathbb{1}(A)$ represents an indicator function that is 1 if event A occurs and 0 otherwise

Sample auxiliary variables ζ and η:

$\zeta \sim \text{Bernoulli}\left(\frac{\bar{M}_{\cdot,\cdot}}{\bar{M}_{\cdot,\cdot}+\gamma}\right)$

$\eta \sim \text{Beta}\left(\gamma + 1, \bar{M}_{\cdot,\cdot}\right)$

Then sample γ as follows:

$\gamma \sim \text{Gamma}\left(A_{\gamma,a} + \bar{K} - \zeta, A_{\gamma,b} - \log \eta\right)$

If not provided as a fixed value, sample ρ:

$\rho \sim \text{Beta}\left(\sum_{u=1}^{S} W_{u,\cdot} + A_{\rho,c}, M_{\cdot,\cdot} - \sum_{u=1}^{S} W_{u,\cdot} + A_{\rho,d}\right)$

Set variables:

$\alpha \leftarrow (\alpha + \kappa)(1 - \rho)$

$\kappa \leftarrow (\alpha + \kappa)\rho$

return α, κ, γ

References

[1] Scheinin, I. *et al.* DNA copy number analysis of fresh and formalin-fixed specimens by shallow whole-genome sequencing with identification and exclusion of problematic regions in the genome assembly. *Genome Res.* **24**, 2022–2032 (2014). DOI 10.1101/gr.175141.114.Freely.
